# Cryo-EM analysis reveals human SID-1 transmembrane family member 1 dynamics underlying lipid hydrolytic activity

**DOI:** 10.1038/s42003-024-06346-8

**Published:** 2024-05-29

**Authors:** Yoshinori Hirano, Umeharu Ohto, Ikuyo Ichi, Ryota Sato, Kensuke Miyake, Toshiyuki Shimizu

**Affiliations:** 1 Graduate School of Pharmaceutical Sciences, The University of Tokyo, 7-3-1 Hongo, Bunkyo-ku, Tokyo 113-0033 Japan; 2 Natural Science Division, Ochanomizu University, Bunkyo-ku, Tokyo 112-8610 Japan; 3 Institute for Human Life Innovation, Faculty of Core Research, Ochanomizu University, Bunkyo-ku, Tokyo 112-8610 Japan; 4 Division of Innate Immunity, Department of Microbiology and Immunology, The Institute of Medical Science, The University of Tokyo, 4-6-1 Shirokanedai, Minato-ku, Tokyo 108-8639 Japan

**Keywords:** Cryoelectron microscopy, Membrane proteins

## Abstract

Two mammalian homologs of systemic RNA interference defective protein 1 (SID-1) (SIDT1/2) are suggested to function as double-stranded RNA (dsRNA) transporters for extracellular dsRNA uptake or for release of incorporated dsRNA from lysosome to cytoplasm. SIDT1/2 is also suggested to be involved in cholesterol transport and lipid metabolism. Here, we determine the cryo-electron microscopy structures of human SIDT1, homodimer in a side-by-side arrangement, with two distinct conformations, the cholesterol-bound form and the unbound form. Our structures reveal that the membrane-spanning region of SIDT1 harbors conserved histidine and aspartate residues coordinating to putative zinc ion, in a structurally similar manner to alkaline ceramidases or adiponectin receptors that require zinc for ceramidase activity. We identify that SIDT1 has a ceramidase activity that is attenuated by cholesterol binding. Observations from two structures suggest that cholesterol molecules serve as allosteric regulator that binds the transmembrane region of SIDT1 and induces the conformation change and the reorientation of the catalytic residues. This study represents a contribution to the elucidation of the cholesterol-mediated mechanisms of lipid hydrolytic activity and RNA transport in the SID-1 family proteins.

## Abstract (web summary)

Structural and biochemical analysis of double-stranded RNA transporter SIDT1 reveals the conformational change associated with cholesterol binding and the causal relationship between pH, RNA binding, and ceramidase activity.

## Introduction

The extracellular RNAs in mammalian body play important roles in cell-cell communication, virus-host interactions, and potent RNA therapeutics^1,2^. Viral double-stranded RNA (dsRNA) triggers an antiviral defense system in vertebrates through the activation of innate immunity. Synthetic small interfering (si)RNAs and micro RNAs (miRNAs) induce evolutionary conserved RNA interference (RNAi), which results in posttranscriptional gene silencing. These RNAs have been suggested to exert their functions in a non-cell autonomous manner through secretion from host cells or exogenous supply and uptake by recipient cells^1,2^. However, dsRNA uptake mechanism is largely unknown.

RNAi initiated by injection of dsRNA is initially found in *Caenorhabditis elegans* (*C. elegans*)^3^. In *C. elegans*, the effect of RNAi spreads from the introduced cells or tissues throughout the organism and its progeny. The *C. elegans* systemic RNAi defective protein 1 (SID-1) has been identified as a gene required for systemic but not cell-autonomous RNAi^4^. SID-1 serves as a dsRNA transporter or channel for uptake into cells in an energy independent manner^5–7^. SID-1 protein is evolutionary conserved although systemic RNAi phenomena are not apparent in mammals, which implies the functional divergence. In mammals, SID-1 homologs SIDT1 and SIDT2 have an ability to transport dsRNA^8–12^. SIDT2 is expressed on endolysosomal membranes to release internalized poly(I:C), a synthetic analog of viral dsRNA, to cytoplasm, which is known as endosomal escape. The released dsRNA activates innate immunity through RIG-I like receptor pathway. Sidt2^-/-^ knockout mice exposed to Encephalomyocarditis virus (EMCV), or herpes simplex virus 1 (HSV-1) show impaired production of inflammatory cytokines and reduced survival^10^. SIDT1 can also transport incorporated internalized poly(I:C) from endolysosome to cytoplasm in the cell-based assay, but Sidt1 knockout mice exposed to EMCV or HSV-1 survive normally, suggesting that SIDT1 contributes less to the activation of innate immunity^11^. SIDT2 is ubiquitously expressed but SIDT1 is mainly expressed in the lymphoid lineage and seems to have a role in the anti-viral IFN-I responses in vivo. A recent report suggests that SIDT1 plays a role in type I interferon response to nucleic acids in plasmacytoid dendritic cells^13^. SIDT1 is also required for cellular uptake of cholesterol conjugated siRNAs in liver HepG2 cells^14^. Moreover, SIDT1 transport miRNAs miR-21 of which is an oncogenic miRNA to mediate chemoresistance in human adenocarcinoma^9^. SIDT1 expressed in gastric pit cells of the stomach is required for the absorption of dietary miRNAs. The stomach is the primary site for dietary miRNA absorption, which is dramatically attenuated in the stomachs of SIDT1-deficient mice^12^. The uptake of miRNAs or double-stranded miRNA mimics by stomach cells are increased in acidic condition, which is not affected in sidt1^-/-^ cells.

Studies from another perspective that SIDT1/SIDT2 shares sequence similarity with *C. elegans* CHUP-1 mediating dietary cholesterol uptake^15^ suggest that mammalian SIDT1/SIDT2 is involved in cholesterol transport. CRAC motif, an α-helical cholesterol binding motif observed in transmembrane proteins, is found in SIDT1/SIDT2 and binds to cholesterol and co-localizes with cholesterol in intracellular compartment. Cells overexpressing SIDT1 enhances cholesterol uptake while the cholesterol reduction in cells induces the relocalization of SIDT1 to the plasma membrane in clathrin dependent manner^16^. Additionally, CHUP-1 is suggested to play a role in protective immune response by linking cholesterol metabolism and the immune response^17^.

The structure of SIDT2 has been recently reported^18^, providing the architecture of SID-1 family protein. But cholesterol-mediated regulation mechanism remains unknown. Our structural work revealed two distinct conformations, the cholesterol-bound form and the unbound form. Cholesterol molecules binds the transmembrane region of SIDT1 and reorient catalytic residues accompanied with a large conformational change of transmembrane helices, suggesting that cholesterol molecule serve as allosteric regulator. Moreover, we identified dsRNA binding site by structure-based mutational studies.

## Results

### Structure determination of two distinct conformations of SIDT1 dependent on pH

**Fig. 1:**
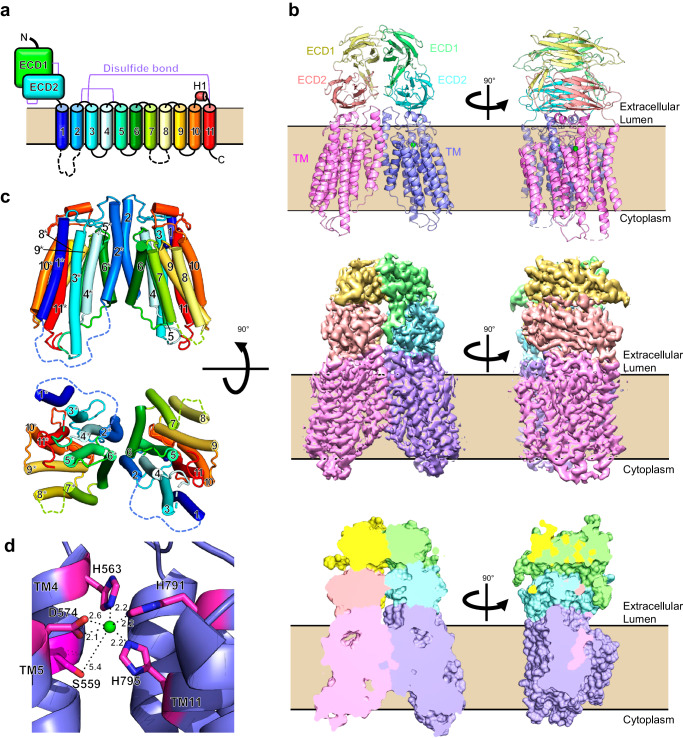
Structure of the human SIDT1 homodimer in cholesterol-bound form. **a** A schematic representation of SIDT1. SIDT1 comprises N-terminal extracellular region with two domains (ECD1, ECD2) and following transmembrane segments which are sequentially numbered from 1 to 11 and colored from blue to red. **b** The ribbon model (top panel), cryo-EM map (middle panel) and cross section view (bottom panel) of the overall structure of hSIDT1 homodimer. The ECD1, ECD2, and TM of one protomer are shown in yellow, salmon and magenta, respectively while those of the other protomer are shown in green, cyan, and purple, respectively. Zinc ions are shown as green spheres. **c** The structure of the membrane-spanning region shown from the side (top panel) and cytoplasmic side (bottom panel). Colors and numbers are as in a, but * is added to the number of the other protomer for clarity. Two cytoplasmic loops, TM1-TM2 and TM7-TM8 (dotted line) are largely disordered. **d** A zinc ion (green sphere) is coordinated by three histidine residues and an aspartate residue in each hSIDT1 protomer. Distances (Å) between Zn^2+^ and its coordinating atoms are indicated.

The full length of human SIDT1 (hSIDT1) (Fig. 1a) was purified from mammalian cells and subjected to cryo-electron microscopy (cryo-EM) single particle analysis for structure determination. SIDT1 embedded in a GDN micelle exhibited the 2D averages of relatively resolved transmembrane helices and we successfully generated a reconstruction of hSIDT1 to an overall resolution of 2.77 Å (Supplementary Fig. 2). The lipid-like densities were inserted at the cytoplasmic side of TM region. These densities were fitted to cholesterol molecules (Supplementary Fig. 3) and Mass spectrometry analysis identified cholesterol in purified hSIDT1 (Supplementary Fig. 4). Thus, this structure is referred to as the cholesterol-bound form.

To clarify the dsRNA recognition by SIDT1, cryo-EM analysis was performed in the presence of dsRNA. Although clear densities corresponding to dsRNA were not observed, the cryo-EM structures of hSIDT1 in the presence of 25 bp dsRNA at pH5.0 were interestingly converged to 2 classes; one class is essentially same as the structure in the absence of dsRNA and similarly binds to cholesterol, while the other class has different conformation of the transmembrane (TM) region with no cholesterol binding (Supplementary Figs. 5, 6). To clarify whether the conformational change is caused by low pH or RNA binding, we have determined the structure of SIDT1 at low pH (pH 5.0) without RNA. At low pH (pH 5.0) conditions, the structures of SIDT1 were converged to 2 classes (cholesterol-bound and cholesterol-unbound forms) as essentially the same as those in the presence of 25 bp dsRNA at pH5.0 (Supplementary Fig. 7). The density of cholesterol was observed in both presence and absence of RNA (Supplementary Figs. 5, 7). Thus, the observed conformational change has been suggested to be caused by low pH. Thereafter, we will describe the structure (cholesterol-bound or cholesterol-unbound form) with the highest resolution.

### Overall structure of the cholesterol-bound form of human SIDT1

SIDT1 is assembled as a homodimer (Fig. 1b). The N-terminal extracellular region of hSIDT1 contains a disordered region (residues 1–43), followed by two extracellular domains (ECD1 and ECD2) arranged in a β-sandwich fold with two four-stranded β-sheets. ECD1 contains an extra β-hairpin between β5- and β8-strands, which contacts with ECD2 (Fig. 1b, Supplementary Fig. 8). The N-glycan densities are observed to be attached to four N-glycosylation sites of ECD1 (Asn57, Asn67, Asn83, and Asn136). The inter-domain disulfide bond (Cys130-Cys222) and the intra-domain disulfide bond in ECD2 (Cys212-Cys271) are formed, of which cysteine residues are conserved in vertebrate SID-1 family proteins, suggesting the evolutionary conserved contribution of disulfide bonds for the stabilization of inter-domain orientation and structural rigidity (Supplementary Figs. 1, 8).

The membrane-spanning region is clearly resolved sufficiently to model all 11 TM helices de novo (Fig. 1b, c, Supplementary Fig. 3). The cytoplasmic long TM1-TM2 and TM7-TM8 loops are largely invisible in the cryo-EM map. The extracellular loops, TM4-TM5 loop, and TM10-TM11 loop, are linked to TM2-TM3 loop by disulfide bonds (Cys479-Cys565 and Cys485-Cys782) (Supplementary Fig. 8). The short helix H1 located in TM10-TM11 loop packs with ECD2 along with TM2-TM3 loop. On the extracellular side of the TM helices, we detected density that would be assignable to Zn^2+^, being tetrahedrally coordinated with His563, Asp574, His791 and His795 (Fig. 1d, Supplementary Fig. 3). Residues coordinating Zn^2+^ ion and forming disulfide bonds are completely conserved in SID-1 family proteins (Supplementary Fig. 1). Interestingly, mutations introduced in Cys464 and His740 of *C. elegans* SID-1 (equivalent to Cys479 and His791 of hSIDT1, respectively) have been shown to strongly attenuate environmental RNAi by ingested dsRNA^19^, suggesting the functional importance of those conserved residues beyond species. This “cholesterol-bound form” of hSIDT1 has no tunnels for nucleotides to pass through within a single protomer or at the dimer interface (Fig. 1b).

Structural similarity analysis using the DALI server^20^ suggested that the membrane-spanning region of SIDT1/2 has no similarities to known structures. However, a deep sequence similarity search previously suggested that SID-1 family is a member of a diverse superfamily of putative metal-dependent transmembrane hydrolases including 7 TM protein alkaline ceramidases (ACERs) and adiponectin receptors (ADIPORs) before their structure determination, mainly based on the conservation of metal-ion coordinating residues^21^. Although SIDT1 contains 11 TMs which is more than 7 TMs observed in the structures of ACERs and ADIPORs^22–24^, structure superposition focused on helices containing the conserved metal-ion coordinating residues surprisingly revealed that membrane spanning region of SIDT1 shares structural similarity with ACER3 and ADIPORs (Supplementary Fig. 9). Out of 11 TMs of SIDT1, 7 TMs except for TM1, TM2, TM7 and TM8 are moderately fitted to ACER3 and ADIPOR2 with a root mean squared deviation (RMSD) of 3 to 4 Å. These structural features raise the possibility that SIDT1 has a ceramidase activity, which will be described later together with the structure of cholesterol-unbound form of SIDT1.

### Dimer interface of the cholesterol-bound form

**Fig. 2:**
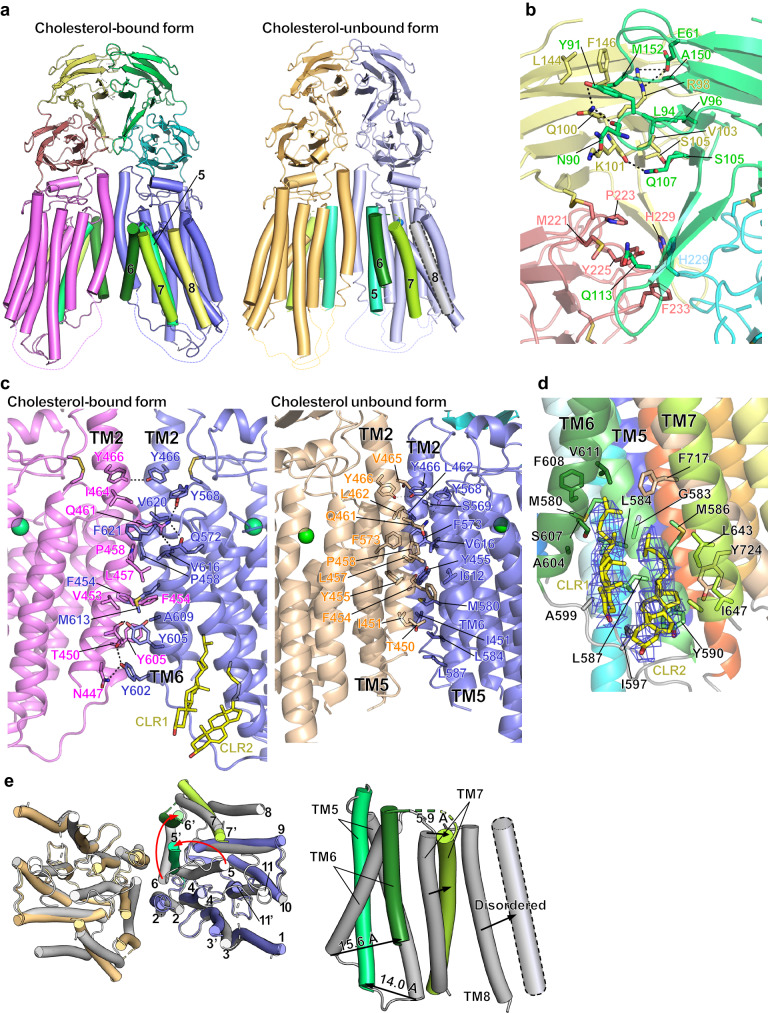
The conformational interconversion of hSIDT1 upon cholesterol binding. **a** The overall structures of hSIDT1 cholesterol-bound form and cholesterol-unbound form. TM5, 6, 7, and 8 which undergo a large conformational change are highlighted. The interfaces in the extracellular region of the cholesterol-bound form (**b**) and transmembrane region (**c**) are shown. The interface in the extracellular region of the cholesterol-unbound form is essentially same as the cholesterol-bound form. Residues which mediate polar and non-polar interactions are shown. Hydrogen bonds are shown as dashed lines. Since SIDT1 forms a C2 symmetric homodimer, only half of the interactions of the dimer interface along the symmetry axis are shown for clarity. **d** Cholesterol molecules (yellow) bind the pocket formed by TM5, TM6, and TM7 of the cholesterol-bound form, which stabilizes the dimer interface. **e** Structural comparison between cholesterol-bound form (gray) and open form.

SIDT1 forms a side-by-side homodimer along the C2 symmetry axis, which is mediated by extensive interactions via several surfaces (Fig. 2a). On the extracellular side, ECD1 contains salt bridges or hydrogen bonds, while in ECD2, the β14-β15 loops of both protomers contact each other primarily through non-polar interactions (Fig. 2b). In the TM region, TM2 and TM6 of both protomers pack with each other, incorporating hydrophobic interactions in the center of the membrane region and hydrogen bonds at the top and bottom of the membrane region (Fig. 2c). These residues are highly conserved in mammalian SID-1 family proteins although it is less conserved in *C. elegans* SID-1 (Supplementary Fig. 1).

Several lipid-like elongated densities were observed in the transmembrane region of the cholesterol-bound form. The two densities run parallel to each other in a small concave formed by TM5, TM6, and TM7 at the intracellular side, which can be modeled as cholesterol molecules (Fig. 2d). Since we did not add cholesterol during purification, endogenous cholesterol is assumed to be co-purified with SIDT1. The putative cholesterol density which is better resolved than the other (CLR1) is located on the opposite of the homo-dimer interface of TM5 and fills the space between TM5 and TM6. Another cholesterol molecule (CLR2) is packed with TM5 and TM7. These cholesterol molecules were surrounded by hydrophobic residues, most of which are conserved in mammalian SID-1 family proteins, suggesting that the cholesterols serve as molecular glue that stabilizes the conformation of TM5, TM6, and TM7.SIDT1 is previously reported to bind cholesterol via CRAC motif^16^ characterized by the sequence (L/V)-X_1−5_-(Y)-X_1−5_-(K/R)^25^. Two CRAC motifs identified in SIDT1by the previous in silico analysis; one locates at the extracellular region and the other locates at the TM region, of which the one in the TM region (residues 633-659) is indicated to be important for cholesterol binding^16^. In our cryo-EM map, the first part of the residues 633-659 in SIDT1 forming TM7 interacts with one of two observed cholesterol molecules (CLR2), and the rest of the region is disordered. However, we found that another CRAC motif in TM5 which is not indicated previously interacts with cholesterol molecules more extensively than TM7 (Fig. 2d, Supplementary Fig. 1).

### Conformational flexibility associated with cholesterol-binding

In the cholesterol-unbound form, the transmembrane region undergoes a large conformational change while the extracellular region is well fitted to that of the cholesterol-bound form structure (RMSD of 0.3 Å for Cα atoms) (Fig. 2a, Supplementary Fig. 10). In the cholesterol-unbound form, TM5 rotates about 25° and its cytoplasmic side greatly moves from inside the membrane spanning region to the dimer interface (14 Å shift of the Cα atom of Thr592), which resulted in the interaction with TM2 of the other protomer (Fig. 2e). TM6 which interacts with TM2 in the cholesterol-bound form is pushed out (15.6 Å shift of the Cα atom of Tyr605) by the TM5 rotational motion and its N-terminal region is partially disordered. These conformational changes of transmembrane region are primarily caused by helix rotation, resulting in shifts of cytoplasmic side with less changes in the extracellular side. The exception is TM7, where the extracellular side is shifted (5.9 Å shift of the Cα atom of Trp627) and TM8 is almost disordered (Fig. 2e). Of note, despite the transmembrane region undergoes a large conformational change, the homodimer is maintained in the cholesterol-unbound form by the alternative interface in which TM5 displaces TM6 in the cholesterol-bound form. The displaced interface is mainly formed by non-polar interactions, which brings the dimer interface of 2,622 Å^2^ as large as that of 2,656 Å^2^ in the cholesterol-bound form (Fig. 2c). In the cholesterol-unbound form, no lipid-like density was observed between TM5 and TM6, which suggests that cholesterol stabilize the cholesterol-bound form structure through binding to the concave between TM5 and TM6.

**Fig. 3:**
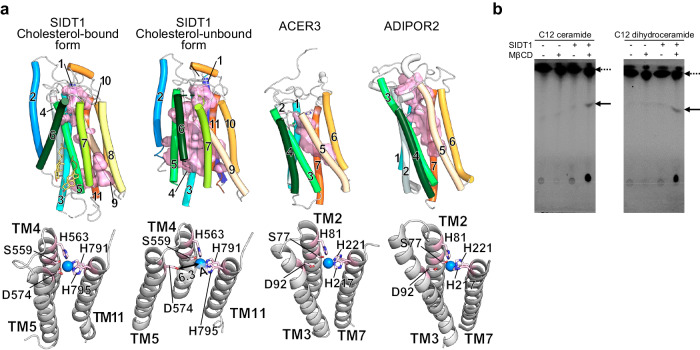
The cholesterol-bound and unbound state of transmembrane tunnel structures of SIDT1 and their structural comparison. **a** In theSIDT1cholesterol-bound form, TM5 interrupts the transmembrane tunnel while in its cholesterol-unbound form, transmembrane tunnel is formed. The TM domain of SIDT1 is similar to those of Alkaline ceramidase 3 (ACER3, PDB:6G7O) and Adiponectin receptor 2 (ADIPOR2, PDBID:5LX9), in which the substrate binding pockets are partially overlapped with the tunnel of SIDT1(shown as pink surface model, upper panel). The catalytic site of hSIDT1, ACER3 or ADIPOR2 (bottom panel). The zinc ion and catalytic residues are shown as sphere and stick model, respectively. **b** Ceramidase assay using C12 NBD ceramide or C12 NBD dihydroceramide in the absence or presence of methyl β-cyclodextrin. Arrows indicate C12 NBD dodecanoic acid, a product of ceramidase reaction while broken arrows indicate ceramides used as substrates in this study.

Interestingly, a tunnel is found to be formed in the transmembrane region in the cholesterol-unbound form structure by the conformational change in the transmembrane region (Fig. 3a). This tunnel is surrounded by TM4, TM5, TM9, TM10, and TM11 and penetrates the transmembrane region by passing through beside the zinc ion. TM8 is supposed to be located outside TM7 according to the weak density although TM8 is disordered in the cryo-EM map of the cholesterol-unbound form, which strongly indicates that TM8 do not intercept the tunnel. This tunnel has a maximum diameter of ~5.0 Å and is ~2.2 Å at its narrowest area, which indicates that the pore size is not acceptable for transport of dsRNA.

The tunnel of the cholesterol-unbound form is partly overlaps with the substrate binding pocket in ACER3. In the catalytic mechanism of ACER3, Zn^2+^ is directly coordinated by three His residues and a water molecule that forms hydrogen bonding with Asp, in which the activated water molecule undergoes nucleophilic attack on the ceramide amide bond. Additionally, Ser residue is predicted to form hydrogen bonding with carbonyl group from the amide bond of ceramide and stabilize the oxyanion hole in the transition state (Fig. 3a). As mentioned above, in the cholesterol-bound form of SIDT1, Zn^2+^ is coordinated by the conserved three His residues and Asp residue, in which there is no space for a water molecule which is activated through the coordination with Zn^2+^. On the other hand, in the cholesterol-unbound form, the carboxyl group of Asp574 is 6.3 Å away from Zn^2+^, which is suitable to place a water molecule between them. These structural features raise the possibility that SIDT1 has a ceramidase activity in a conformation dependent manner. We performed ceramidase assay using fluorescent labeled ceramide or dihydroceramide. Interestingly, SIDT1 exhibited a ceramidase activity only in the presence of methyl β-cyclodextrin, which is often used for the depletion of cholesterol from membranes (Fig. 3b). Overall, our results suggest that SIDT1 endows a hydrolase activity which is allosterically inhibited by cholesterol although no endogenous substrates have been identified.

### SIDT1 binds dsRNA in a pH-dependent manner

**Fig. 4:**
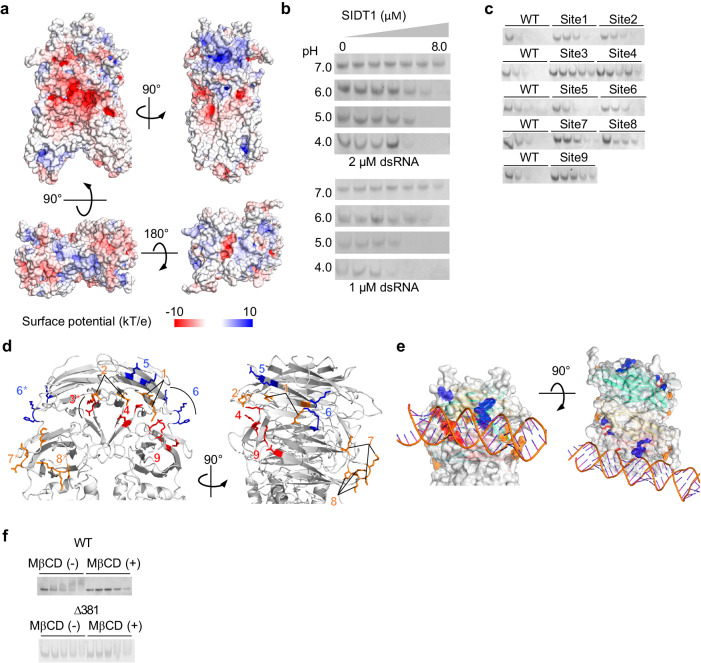
Low pH-dependent dsRNA binding of SIDT1. **a** electrostatic surface potential of hSIDT1. Negative charges are shown in red and positive charges are shown in blue. **b** EMSA using full length hSIDT1 and 46 bp dsRNA at different pH. Unshifted band corresponding to unbound dsRNA was monitored to estimate dsRNA binding with SIDT1 since hSIDT1-dsRNA complex less migrated. **c** EMSA using SIDT1 mutants at pH 5.0. Increasing concentration of hSIDT1 (1, 2, 3, 4, 5 μM) were incubated with 1 μM of dsRNA and subjected to electrophoresis. **d** mutated sites are mapped on the structure of SIDT1. Mutants which greatly (red), modestly (orange) or less (blue) decreased the dsRNA binding are mapped on the molecular surface of SIDT1. Asterisks indicate site of the other protomer. **e** dsRNA is placed across the lateral side of SIDT1 ECD. Color schemes are the same as (**d**). **f** as in (**c**), EMSA using SIDT1 mutants in the presence or absence of MβCD were incubated with 1 μM of dsRNA and subjected to electrophoresis.

The extracellular region of SID-1 family proteins has been shown to bind dsRNA^26^. We performed electrophoretic mobility shift assay (EMSA) at pH 7.0 using full length hSIDT1 and observed a concentration-dependent dsRNA shift, confirming the interaction between hSIDT1 and dsRNA (Fig. 4b). Structural analysis of hSIDT1 revealed that the extracellular region of hSIDT1 homodimer exhibits a positively charged surface on the lateral side, but a strongly negatively charged surface in the anterior side (Fig. 4a). Since this negative charge might be unfavorable for dsRNA-binding due to its electrostatic repulsion with the negatively charged phosphate group of dsRNA, we speculated that the dsRNA interaction might be pH-dependent and performed EMSA at different pH (4.0–7.0) to verify the pH dependence of dsRNA binding. Unshifted band corresponding to unbound dsRNA was monitored to estimate dsRNA binding with SIDT1. Interestingly, unbound dsRNA was diminished with lower concentration of SIDT1 as pH decreased, strongly suggesting that SIDT1 binds dsRNA in a low-pH dependent manner (Fig. 4b). The low-pH dependent dsRNA binding of SIDT1 is consistent with the previous observations that microRNA or double-stranded microRNA mimic uptake in mouse primary gastric epithelial cells (PGECs) is SIDT1 and low-pH dependent^12^.

The previous genetic screens in *C. elegans* have identified several loss-of-function mutations in SID-1^14,19^. As the result of mapping these mutations on our hSIDT1 structure, most residues are buried inside SIDT1 to support the protein folding while Arg172 (corresponding to His168 in hSIDT1) is exposed to solvent (Supplementary Fig. 11). Moreover, low sequence identities between SID-1 and mammalian SIDTs hampered the identification of dsRNA binding site. To estimate the dsRNA binding site of SIDT1, we have applied alanine scanning mutagenesis to mutate residues with polar groups in the extracellular region to alanine and monitored their effects on dsRNA binding by EMSAs (Fig. 4c). The wild-type SIDT1 required 3–4 equimolar amounts to saturate dsRNA binding. The nine mutants (site1-site9), containing a total of 22 mutations, were classified into three classes based on how much they attenuated dsRNA binding of wild-type SIDT1; large, modest, or less effect. The mutants site3, site4, and site9 have large effects and the mutants site1, site2, site7, and site8 have modest effects while the mutants site5 and site6 have less effects. Those mutants are mapped on the structure of SIDT1 (Fig. 4d). Among mutations that reduce dsRNA binding of SIDT1, site1 (Arg79, Tyr81), site2 (Glu87, Asp90), site4 (Asn121, Gln123) and site9 (Gln288, Lys290, Asn292) are located in close proximity to the lateral side of the extracellular region, suggesting that these residues are involved in a dsRNA binding. Site7 (Lys192, Lys195, Asp196) and site8 (Lys250, Lys251, Asp252) are distant from those sites and near the transmembrane region. Placing a liner dsRNA on a line connecting these two regions allows a dsRNA to be placed across the lateral side of SIDT1 ECD with only a slight steric clash (Fig. 4e).

Additionally, in SIDT2, arginine-rich motif mostly conserved in SIDT1 in the TM1-TM2 loop, which is largely disordered in our cryo-EM map (Supplementary Fig. 12a), is reported to bind RNA, DNA, and oligonucleotide^27–29^. Hence an EMSA assay was performed to verify the contribution of the TM1-TM2 loop to dsRNA binding of SIDT1. The deletion of residues of the TM1-TM2 loop (residues 391–442 and 381–442) decreased dsRNA binding of SIDT1, suggesting that the TM1-TM2 loop of SIDT1 binds to dsRNA (Supplementary Fig. 12b). To examine the cholesterol binding effect for dsRNA binding, we conducted the EMSA assay using wild type SIDT1 or the SIDT1 mutant (Δ381) deleting the cytoplasmic dsRNA binding region. The dsRNA binding of wild-type SIDT1 was reduced in the presence of MβCD compared to the absence of MβCD, suggesting that cholesterol removal weakens the dsRNA binding of hSIDT1 (Fig. 4f). On the other hand, the dsRNA binding of this mutant was not affected by the presence or absence of MβCD in the EMSA assay (Fig. 4f), strongly suggesting that cholesterol binding affect the RNA binding to the cytoplasmic region.

## Discussion

Our biochemical and structural analyses have identified the dsRNA binding site at the lateral side of extracellular region (Fig. 4e), the surface of which is positively charged, but the anterior side is strongly negatively charged (Fig. 4a). Unlike hSIDT1, the predicted structure of *C. elegans* SID-1 by alphafold2 has a strong positive charge on the lateral side and no negative charge on the anterior side (Supplementary Fig. 13). This difference in surface charge may be one of the reasons for the difference in affinity, as it was previously reported that the affinity of SIDT1 to dsRNA (500 bp or 700 bp long chain) is about 1/5 that of SID-1 in EMSA using extracellular domains^26^. Moreover, we have revealed that SIDT1 binds dsRNA in a low pH dependent manner. The low-pH-dependent dsRNA binding is consistent with a physiological function of SIDT1, since the extracellular domain of SIDT1 is exposed to an acidic environment outside the cell membrane of gastric pit cells of the stomach or inside lysosomal membranes^12^. Bound dsRNA can spontaneously dissociate from SIDT1 in a neutral pH of the cytoplasm, which might work in the releasing process.

Recently, the structure of human SIDT2 has been reported^18^. The dimer architecture of human SIDT2 mediated by the interaction between TM2 and TM6 in the TM region is similar to the cholesterol-bound form of SIDT1 although the ECD1 of SIDT1 lacks an N-terminal β-strand observed in SIDT2 (Supplementary Fig. 14a). Strikingly, the structure of SIDT2 did not contain cholesterol molecules. Compared with SIDT2, the concave between TM6 and TM7 of SIDT1 is forced to expand by cholesterol binding, which brings the TM6 longer and closer to dimer-interface. Thus, the interfacing TM2 of the other protomer adopts a longer helix to extensively interact with the TM6. Additionally, the putative Zn^2+^ coordination by SIDT2 differs greatly from that of the cholesterol-bound form of SIDT1. As described above, SIDT1 Asp574 coordinates Zn^2+^ together with the conserved triad of histidine residues, while in SIDT2, the corresponding residues Asp579 is as far from Zn^2+^ as the corresponding aspartate residues in ACERs, ADIPORs or the cholesterol-unbound form of SIDT1 (Fig. 3a Supplementary Fig. 14b). Consistent with the structural insight, it is revealed that SIDT2 has a lipid hydrolase activity for ceramide^18^, which is first evidence that SID-1 family proteins have a hydrolase activity. We here showed that SIDT1 also harbors a ceramidase activity to ceramide and dihydroceramide although the physiological substrate is not identified. Importantly, the cholesterol depletion by MβCD enhanced the hydrolase activity of SIDT1 (Fig. 3b), which suggests that cholesterol inhibits SIDT1 hydrolase activity. Furthermore, conformational interconversion of the TM region likely associated with cholesterol binding observed in our cryo-EM study provides the structural insight into the action of cholesterol as an allosteric regulator, which induces the rearrangement of TM region mainly in the TM5-TM8 segment to lock Asp574 and excludes a water molecule from zinc ion coordination, probably resulting in the inhibition of the hydrolase activity. Recently, the cholesterol-unbound structures of human SIDT1 have been reported^30,31^, in which the structures of wild-type SIDT1 are similar to our structure of cholesterol-unbound form and poses a lipid hydrolytic activity while the structure of hSIDT1 E555Q with reduced lipid hydrolytic activity is similar to our cholesterol-bound form. These results are consistent with the conformation-dependent lipid hydrolase activity of SIDT1 which is likely driven by cholesterol-binding in our study although it is not clear that the mutation introduced in Glu555, which is located near Zn^2+^, induces similar conformation change to cholesterol-binding. Our results highlight wild-type SIDT1 dynamics underlying lipid hydrolytic activity without introducing mutations. In the crystal structure of ADIPOR1, closed- and open-form are observed, mainly due to the difference of the TM5 conformation (corresponding to the TM9 in SIDT1)^24,32^. The dynamic equilibrium between open and closed conformation in ADIPORs hypothesized to regulate the substrate binding and the product release^24^. Thus, the open-closed conformational change of ADIPORs is extremely different from SIDT1, indicating that SIDT1 develops a unique catalytic regulation mechanism. Moreover, the conformational change of SIDT1 observed in our structures may reflect the effect of releasing cholesterol in the transport process since SIDT1 and SIDT2 have been reported to be involved in cholesterol transport^16^. Further study is required to verify whether this mechanism is conserved in other SID-1 family proteins.

We have revealed that the conformational change likely associated with cholesterol binding and dsRNA binding to hSIDT1 are pH dependent, but their relationship is not clear. In the EMSA assay, cholesterol removal weakened the dsRNA binding of hSIDT1 (Fig. 4f). hSIDT1 harbors two dsRNA binding sites that are located in the extracellular region and the cytoplasmic region. The dsRNA binding to the extracellular region is unlikely to be affected by the cholesterol binding since cholesterol binding does not induce conformational change in the extracellular region, but in the TM region. Indeed, the dsRNA binding of the mutant deleting the cytoplasmic dsRNA binding region (residues 381-442) was not affected by the presence or absence of MβCD in the EMSA assay (Fig. 4f). The cytoplasmic dsRNA binding region located in TM1-TM2 loop is disordered, which hampered the understanding of conformational change. However, TM2 itself undergoes the conformational change and interacts with TM5 and TM6 that are involved in the cholesterol binding, which supports the notion that the dsRNA binding to TM1-TM2 loop can be affected by the cholesterol binding.

During the preparation of this manuscript, structures of human SIDT1 and SIDT2 were reported, in which no cholesterol densities were observed but a phosphatidic acid bound as substrate in one of reports. We summarized the samples used for structural studies and compared sample preparation procedures (Supplementary Table 1). Although it is not clear why our SIDT1 sample is co-purified with cholesterol and others were not, the protein purification procedures including solubilizing detergent for protein extraction from cell membranes and/or expression cells are different, which can affect the lipid binding.

The structures of SIDT1 presented here provide insight into the architecture of SID-1 family proteins, homo-dimer state, and the conformational change. The membrane-spanning region of SIDT1 has a conformation-dependent tunnel which partially shares the substrate binding pocket of AdipoRs and ACERs. However, the pore size of the tunnel is not acceptable for dsRNA transport. Our structures revealed that cholesterol molecules bind to TM region of SIDT1 and likely induce the conformation change to interrupt the tunnel. Previous study has reported that sterol molecules bind to SIDT1 and SIDT2, which control the cellular localization^16^. Of note, this study also reported that mixing dsRNA with cholesterol resulted in increased uptake of dsRNA in HEK293 cells overexpressing SIDT1. Taken together, cholesterol might regulate dsRNA transport by SIDT1 although it is not clear how dsRNA transport is linked to the cholesterol-binding induced conformational change. Further structures that capture the progressive states of RNA binding and cytoplasmic release may enable the mechanism of the RNA transport to be determined.

## Methods

### Protein expression and purification

The cDNAs of human SIDT1 were obtained from a commercial source (Dharmacon). DNA fragments were amplified by the polymerase chain reaction (PCR) and cloned into the pEZT-BM vector. These proteins were fused with a C-terminal TEV protease cleavage site, FLAG tag and decahistidine tag. We designated the pEZT-BM vector to incorporate TAR-Tat element derived from pHEK293 ultra vector (TaKaRa) to enhance protein expression. All plasmids were verified by DNA sequencing. Baculoviruses were generated in *Spodoptera frugiperda* Sf9 cells using the Bac-to-Bac system (Invitrogen). For protein expression, Expi293F cells (Invitrogen) were cultured in Expi293 expression medium at 37 °C under 8% CO_2_ in a CO_2_ incubator (PHC). When the cell density reached 3.0–6.0 × 10^6^ cells per mL, cells were diluted to 3.0 × 10^6^ cells per mL and P4 virus was added at a final concentration of 7% (v/v). Cells were cultured at 30 °C for 4 days in the presence of 10 mM sodium butyrate or 5 mM sodium valproate to enhance protein expression.

Harvested cells were suspended in buffer A (20 mM Tris-HCl buffer (pH 7.0) containing 150 mM NaCl) and protease inhibitor cocktail, and then disrupted by sonication on ice. After sonication, lysates were solubilized by 1.7% (w/v) digitonin at 4 °C for 1–2 h. After centrifugation at 18,000 rpm for 20 m, the supernatant was collected and loaded on to Anti DYKDDDDK tag Antibody Beads (Fujifilm). After extensively washing with buffer A containing 0.01% GDN proteins were eluted with buffer B (20 mM MES-NaOH (pH6.0) containing 150 mM NaCl and 0.01% GDN101) supplemented with 5 M LiCl. The eluted proteins were further purified by gel filtration chromatography (Superdex 200 increase, GE Healthcare) in buffer B. The peak fractions were concentrated using Amicon Ultra centrifugal filter (100-kDa MW cut-off), frozen in liquid nitrogen, and stored at -80 °C until use.

### Cryo-EM sample preparation and data acquisition

Protein samples were adjusted to 2.5 to 5.0 mg ml^–1^. For sample preparation of SIDT1 in the presence of dsRNA, 40 μM of 25 bp dsRNA (5’-CUGCGGACUAUUUGGCAAAGGAAGC) was mixed with protein samples in a buffer 100 mM sodium acetate (pH5.0), 10 mM MES-NaOH, 75 mM NaCl and 0.005% GDN. Three-microliter aliquots of samples were placed onto a freshly glow-discharged Quantifoil holey carbon grids (R1.2/1.3, Cu, 300 mesh). After 4 s of blotting in 100% humidity at 6 °C, the grid was plunged into liquid ethane using a Vitrobot MkIV (Thermo Fisher Scientific). Cryo-EM data collection was performed using a Titan Krios G4 microscope (Thermo Fisher Scientific), running at 300 kV and equipped with a Gatan Quantum-LS Energy filter (GIF) and a Gatan K3 camera in electron-counting mode, at the Cryo-EM facility of the University of Tokyo, Japan. Imaging was performed at a nominal magnification of ×105,000, which corresponded to a calibrated pixel size of 0.83 Å px^–1^. Each movie was recorded in CDS mode for 5.0 s and subdivided into 64 frames with an accumulated exposure of 65.7 e^–^ per Å^2^ at the specimen. The data were acquired by the image-shift method using the SerialEM software^33^. Cryo-EM data of hSIDT1 at pH 6.0 or in the presence of RNA were analyzed using RELION 3.1^34^. Raw movie stacks were motion-corrected using MotionCor2^35^. The CTF parameters were determined using the CTFFIND4 program^36^. The data processing workflow is summarized in Supplementary Figs. 2, 5. Cryo-EM data of hSIDT1 at pH 5.0 were analyzed by cryoSPARC v.4.4^37^. Raw movie stacks were motion-corrected using patch motion correction. The CTF parameters were determined using patch CTF estimation. The data processing workflow is summarized in Supplementary Fig. 7. The final resolution was estimated by gold-standard Fourier shell correlation (FSC) between the two independently refined half maps (FSC = 0.143)^38^. The atomic model of hSIDT1 was manually accomplished using the Coot program^39^. The built model was refined through alternating cycles using the Coot and PHENIX programs^40^. The refinement statistics are summarized in Table 1.

**Table 1:** Cryo-EM data collection, refinement, and validation statistics

	Human SIDT1 (cholesterol-bound form) PDB 8KCW	Human SIDT1 in the presence of 25 bp dsRNA (cholesterol-unbound form) PDB 8KCX
Data collection and processing		
Magnification	105,000	105,000
Voltage (kV)	300	300
Electron exposure (e^–^/Å^2^)	65.8	61.5
Defocus range (μm)	–0.8 to −1.6	−0.8 to −1.6
Pixel size (Å)	0.83	0.83
No. of micrographs	3297	5002
Symmetry imposed	*C*_2_	*C*_2_
Initial particle images (no.)	2,180,197	2,383,232
Final particle images (no.)	59,745	62,617
Map resolution (Å)	2.77	2.96
FSC threshold	0.143	0.143
Map resolution range (Å)	2.77–10.0	2.96–10.0
Refinement		
Initial model used	de novo	
FSC threshold	0.5	0.5
Correlation coefficient (CCmask)	0.82	0.83
Model resolution range (Å)	2.77–10.0	2.96–10.0
Map sharpening *B* factor (Å^2^)	74.7	74.5
Model composition		
Non-hydrogen atoms	10,484	9486
Protein residues	1252	1142
Glycan residues (NAG, BMA)	20	20
Cholesterols	2	0
Ions (Zn)	2	2
*B* factors (Å^2^)		
Protein	93.0	109.0
Glycan residues (NAG, BMA)	34.3	156.6
Cholesterol	52.4	
Ions (Zn)	129.6	138.8
R.m.s. deviations		
Bond lengths (Å)	0.005	0.004
Bond angles (°)	0.77	0.717
Validation		
MolProbity score	1.47	1.23
Clashscore	5.92	4.56
Poor rotamers (%)	0.18	0.00
Ramachandran plot		
Favored (%)	97.24	98.20
Allowed (%)	2.76	1.80
Disallowed (%)	0.00	0.00

### Structure and sequence comparison

Multiple sequence alignments of the SID-1 family proteins were performed using CLUSTAL Omega. Structure figures were prepared using the PyMOL Molecular Graphics System, Schrödinger, L., & DeLano, W or UCSF Chimera^41^.

### Ceramidase assay

C12 NBD Ceramide (d18:1/12:0) or C12 NBD dihydro Ceramide (d18:0/12:0) was dissolved in chloroform and dried by speed-vac. 1 nmol of NBD ceramide were suspended in the buffer containing 20 mM Tris-HCl (pH 8.0), 150 mM NaCl and 0.01% GDN and sonicated. 8 μg of SIDT1 was added to start the reaction in the presence or absence of methyl β-cyclodextrin (10 mM) and incubated at 37 °C for 3 h. The extraction solvent (chloroform: methanol, 1:1) was added with 3 volumes of reaction mixture to quench the reactions. After centrifugation, the organic phase was collected and dried by speed-vac. 5 μl of each reaction mixture was spotted onto a TLC plate, developed in a solvent system consisting of chloroform, methanol, and 25% ammonium hydroxide (90:30:0.5). The TLC plate was dried and scanned by an imaging system.

### EMSA assay

46 bp dsRNA (5′-AUUCUGCGGAUUAUUUGGCAAAGGAAGCAUUGA-CACAUGCGCCAAU) was used. Increasing concentration of hSIDT1 (0, 0.2, 0.5, 1.0, 2.0, 4.0, 8.0 μM) was incubated with 2 μM or 1 μM of 46 bp dsRNA at 4 °C for 2 h. After incubation, the mixture was subjected to electrophoresis in Tris-Acetate buffer prepared to the indicated pH. Unshifted band corresponding to unbound dsRNA was monitored to estimate dsRNA binding with SIDT1 since hSIDT1-dsRNA complex less migrated. For SIDT1 mutants, EMSA was performed in pH5.0. Increasing concentration of hSIDT1 (1, 2, 3, 4, 5 μM) was incubated with 1 μM of dsRNA and subjected to electrophoresis. Uncropped gel images of EMSA assay are provided in Supplementary Fig. 15.

### Mass spectrometry analysis

Lipids were extracted using the Bligh and Dyer method^42^. For cholesterol analysis, the lipids were saponified at 70 °C for 1 h. Following the saponification, the sample solution was reacted with a trimethylsilylating agent at room temperature for 30 min. The derivatized were quantified by using a GC/MS QP2010 (Shimadzu, Kyoto, Japan) equipped with an SPB-1 fused silica capillary column of 60 m × 0.25 mm and 0.25 μm phase thickness (Supelco Inc., Bellefonte, PA, USA). The temperature program was initiated at 180 °C for 1 min, 20 °C/min at 250 °C, and then 5 °C/min at 290 °C and held for 30 min. The injection temperature was set at 250 °C, the interface at 250 °C, and the ion source adjusted to 200 °C. Quantification was performed using the selected ion monitoring (SIM) mode, with ions observed at m/z 329, 368, and 458 for cholesterol (quantification ions are underlined).

### Reporting summary

Further information on research design is available in the Nature Portfolio Reporting Summary linked to this article.

### Supplementary information

Supplementary information

Description of Additional Supplementary Files

Supplementary Data 1

Reporting Summary

## Data Availability

All data needed to evaluate the conclusions in the paper are present in the paper and/or Extended Data Figures and Table. Additional data and resources related to this paper may be requested from the authors. The cryo-EM maps and related structure coordinates of hSIDT1 cholesterol-unbound form and cholesterol-bound form have been deposited in the EMDB and PDB under accession codes EMD-37113, EMD-37112 and PDB: 8KCX, 8KCW, respectively. The source data behind the graphs in the paper can be found in Supplementary Data 1.
