# Supplementary material for: Cryo-EM analysis reveals human SID-1 transmembrane family member 1 dynamics underlying lipid hydrolytic activity
Source: Commun Biol. 2024 May 29;7:664. doi: 10.1038/s42003-024-06346-8 (PMC11137008; doi:10.1038/s42003-024-06346-8)
Supplement: Supplementary file 1 — Supplementary information [file 42003_2024_6346_MOESM1_ESM.pdf]

Supplementary Information

CryoEM analysis reveals human SID1 transmembrane family member1 dynamics underlying lipid hydrolytic activity

Authors

Yoshinori Hirano[1], Umeharu Ohto[1], Ikuyo Ichi[2,3], Ryota Sato[4], Kensuke Miyake[4], Toshiyuki Shimizu[1]*

Affiliations

[1]Graduate School of Pharmaceutical Sciences, The University of Tokyo, 7-3-1 Hongo, Bunkyo-ku, Tokyo, 113-0033, Japan.

[2]Natural Science Division, Ochanomizu University, Bunkyo-ku, Tokyo, 112-8610, Japan.

[3]Institute for Human Life Innovation, Faculty of Core Research, Ochanomizu University, Bunkyo-ku, Tokyo, 112-8610, Japan.

[4]Division of Innate Immunity, Department of Microbiology and Immunology, The Institute of Medical Science, The University of Tokyo, 4-6-1 Shirokanedai, Minato-ku, Tokyo, 108-8639, Japan.

*Corresponding author:

Email: shimizu@mol.f.u-tokyo.ac.jp (T.S.);

This file includes

Supplementary Figures 1-14

Supplementary Tables 1,2

```
                                                                                β1            β2
hSIDT1 MRGCLRLALLCALPWLLLAASPGHPAKSPRQPPAPRRDPFDAARGADFDHVYSGVVNLSTENIYSFNYTS 70
mSIDT1 MLDCLRLALLCALPWLLRAAVPGHQEEPLAKSAELRRDPRDPARGADFDRVYSGVVSLSTENIYSFNHTS
hSIDT2 -------MFALGLPFLVLLVASVESHLGVLGP------KNVSQKDAEFERTYVDEVNSELVNIYTFNHTV
mSIDT2 -------MIAWRLPLCVLLVASVESHLGALGP------KNVSQKDAEFERTYADDVNSELVNIYTFNHTV
cSID-1 ---MIRVYLI-ILMHLVIGLT—QNNSTTPSPIITSSNSSVLVFEISSKMKMIEKKLE—ANTVHVLRLE

            β3                   β4          β5        β6      β7       β8
hSIDT1 Q—PDQVTAVRVYVNSSS----ENLNYPVLVVVRQQ-KEVLSWQVPLLFQGLYQRSYNYQEVSRTLCPSE 133
mSIDT1 H—PGQVTAVRVHVNSSS----DNLDYPVLVVVRQQ-KEVLSWQVPLLFQGLYQRSYNYQEVSRTLCPSK
hSIDT2 T—RNRTEGVRVSVNVLN----KQKGAPLLFVVRQK-EAVVSFQVPLILRGMFQRKYLYQKVERTLCQPP
mSIDT2 T—RNRTEGVRVSVNVLN----KQKGAPLLFVVRQK-EAVVSFQVPLILRGLYQRKYLYQKVERTLCQPP
cSID-1 LDQSFILDLTKVAAEIVDSSKYSKEDGVILEVTVSNGRDSFLLKLPTVYP-----NLKLYTDGKLLNPLV

                          β9             β10                       β11
hSIDT1 ATNETG----------PLQQLIFVDVASMAPLGAQYKLLVTKLKHFQ------LRTNVAFHFTAS---PS 184
mSIDT1 ATNETG----------PLEQLIFVDVASMAPHGAHYKLLVTKIKHFQ------LPTNVAFYFTAS---PS
hSIDT2 TKNE------------SEIQFFYVDVSTLSPVNTTYQLRVSRMDDFV------LRTGEQFSFNTT---AA
mSIDT2 TKNE------------SEIQFFYVDVSTLSPVNTTYQLRVNRVDNFV------LRTGELFTFNTT---AA
cSID-1 EQDFGAHRKRHRIGDPHFHQNLIVTVQSRLNADIDYRLHVTHLDRAQYDFLKFKTGQTTKTLSNQKLTFV

     β12           β13           β14                           β15      β16
hSIDT1 QPQYFLYKFP-KDVDSVIIKVVSEMAYPCSVVSVQNIMCPVYDLDHN--VEFNGVYQSMTKKAAITLQKK 251
mSIDT1 QPQYFLYKFP-EDVDSVIIKVVSEKAYPCSVVSVQNIMCPVYDLDHN--VEFNGVYQSMTKKAAITLQKK
hSIDT2 QPQYFKYEFP-EGVDSVIVKVTSNKAFPCSVISIQDVLCPVYDLDNN--VAFIGMYQTMTKKAAITVQRK
mSIDT2 QPQYFKYEFP-DGVDSVIVKVTSKKAFPCSVISIQDVLCPVYDLDNN--VAFIGMYQTMTKKAAITVQRK
cSID-1 KPIGFFLNCSEQNISQFHVTLYSED-DICANLITVPANESIYDRSVISDKTHNRRVLSFTKRADIFFTET

        β17                                           β18            TM1
hSIDT1 DFP-GEQFFVVFVIKPEDYACGGSFFIQE---------KENQTWNLQRKKNLEVTIVPSIKESVYVKSSL 311
mSIDT1 DFP-DEQFFVVFVIKPEDYACGGSFSIQE---------NENQTWNLQRSKNLKVTIVPSIKESVYVKSSL
hSIDT2 DFP-SNSFYVVVVVKTEDQACGGSLPFYP-------FAEDEPVDQGHRQKTLSVLVSQAVTSEAYVSGML
mSIDT2 DFP-SNSFYVVVVVKTEDQACGGSLPFYP-------FVEDEPVDQGHRQKTLSVLVSQAVTSEAYVGGML
cSID-1 EISMFKSFRIFVFIAPDDSGCSTNTSRKS----------------FNEKKKISFEFKKLENQSYAVPTAL

hSIDT1 FSVFIFLSFYLGCLLVGFVHYLRFQRKSIDGSFG---SNDGSGNMVASHPIAASTPEGSNYGTIDESSSS 378
mSIDT1 FSIFVFLSFYLGCLLVVLVHHVRFQRKSIDGSFG---SSDGSGNMAVSHPITASTPEGSNYGAIDESSSS
hSIDT2 FCLGIFLSFYLLTVLLACWENWRQKKKTLLVAIDRACPESGHPRVLADSFPGSSPYEGYNYGSFENVSGS
mSIDT2 FCLGIFLSFYLLTVLLACWENWRQRKKTLLLAIDRACPESGHARVLADSFPGSAPYEGYNYGSFENGSGS
cSID-1 -MMIFLTTCLLFLPIVINIIKNSRKLAP-------------SQSNLISFSPVPSEQRDMDL-------SH

hSIDT1 PGRQMSSS---------------DGGPPGQSDTDSSVEESDFDTMPDIESDKNIIRTKMFLYLSDLSRKD 433
mSIDT1 PGRQMSSS---------------DGGQPCHSDTDSSVEESDFDTMPDIESDKNVIRTKMFLYLSDLSRKD
hSIDT2 TDGLVDSAGTGDLSYGYQGRSFEPVGTRPRVDSMSSVEEDDYDTLTDIDSDKNVIRTKQYLYVADLARKD
mSIDT2 TDGLVESAGSGDLSYSYQDRSFDAVGPRPRLDSMSSVEEDDYDTLTDIDSDKNVIRTKQYLCVADLARKD
cSID-1 DEQQNTSS-------------ELENNGEIPAAENQIVEEITAENQETS------------VEEGNREIQV

                         TM2
hSIDT1 RRIVSKKYKIYFWNIITIAVFYALPVIQLVITYQTVVNVTGN---QDICYYNFLCAHPLGVLSAFNNILS 500
mSIDT1 RRIVSKKYKIYFWNIITIAVFYALPVMQLVITYQYVVNVTGN---QDICYYNFLCAHPLGVLSAFNNILS
hSIDT2 KRVLRKKYQIYFWNIATIAVFYALPVVQLVITYQTVVNVTGN---QDICYYNFLCAHPLGNLSAFNNILS
mSIDT2 KRVLRKKYQIYFWNIATIAVFYALPVVQLVITYQTVVNVTGN---QDICYYNFLCAHPLGNLSAFNNILS
cSID-1 KIPLKQDSLSLHGQMLQYPVAIILPVLMHTAIEFHKWTTSTMANRDEMCFHNHACARPLGELRAWNNIIT

            TM3                                   TM4
hSIDT1 NLGHVLLGFLFLLIVLRRDILHRRALEAKDIFAVEYGIPKHFGLFYAMGIALMMEGVLSACYHVCPNYSN 570
mSIDT1 NLGHVLLGFLFLLIVLRRDLLHRRALEAKDIFAMEYGIPKHFGLFYAMGIALMMEGVLSACYHVCPNYSN
hSIDT2 NLGYILLGLLFLLIILQREINHNRALLRNDLCALECGIPKHFGLFYAMGTALMMEGLLSACYHVCPNYTN
mSIDT2 NLGYILLGLLFLLIILQREINHNRALLRNDLYALECGIPKHFGLFYAMGTALMMEGLLSACYHVCPNYTN
cSID-1 NIGYTLYGAIFIVLSICRRGRHEYSHVF----GTYEC----TLLDVTIGVFMVLQSIASATYHICPSDVA
```

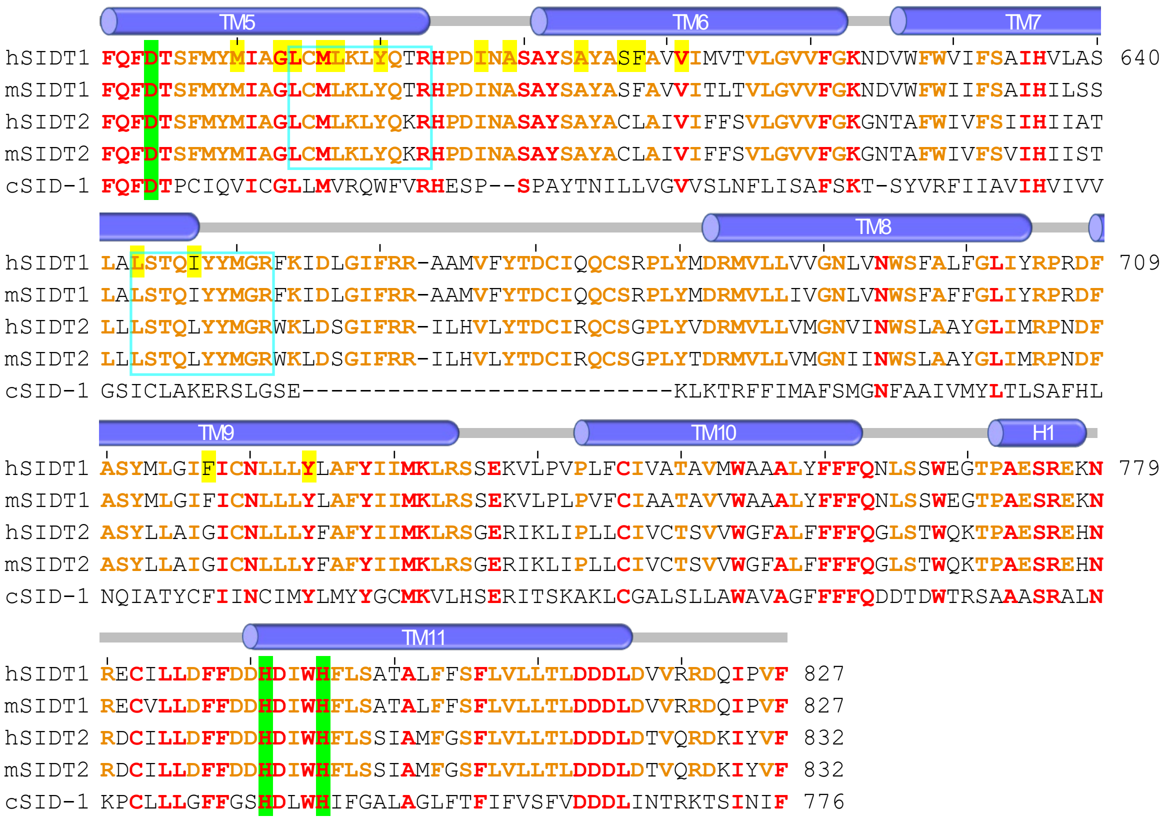

Supplementary Fig. 1 Sequence alignment of SID-1 family proteins.

Sequence alignment of human, mouse SIDT1/ SIDT2 as well as *C. elegans* SID-1were performed using Clustal Omega with a small manual modification. Secondary structures are indicated in arrow-head (β-sheet) or cylinder (helix) above the sequence of human SIDT1. Residues completely conserved in listed SID-1 family proteins are red while the residues conserved in listed mammalian SID-1 family proteins are shown in red and orange, respectively. The catalytic residues are highlighted in green. Residues involved in cholesterol binding is highlighted in yellow. The CRAC motifs in extracellular region (β10), TM5 and TM7 are boxed in cyan.

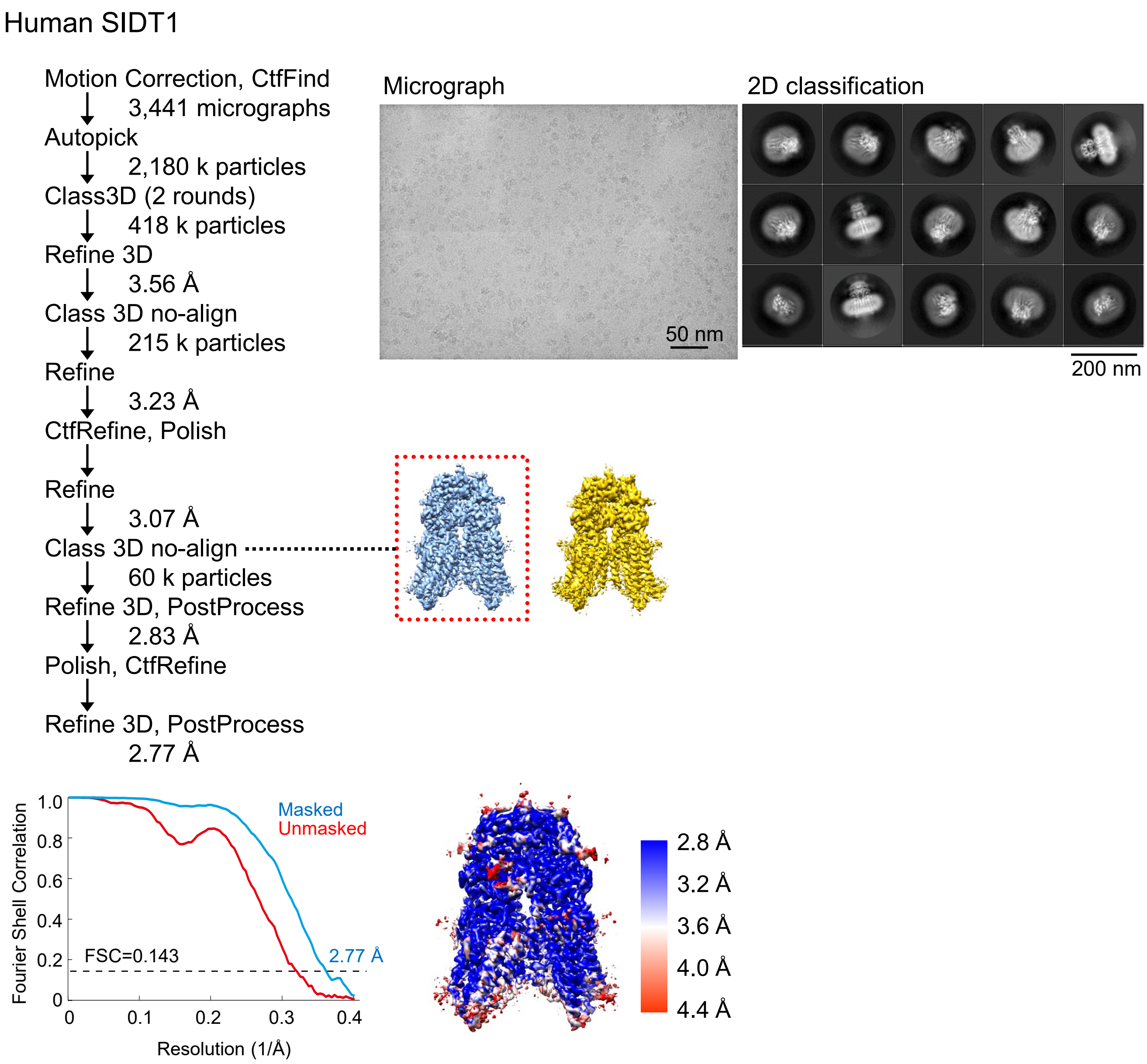

Supplementary Fig. 2 Cryo-EM analysis of human SIDT1.

Data processing workflow of cryo-EM analysis of human SIDT1. Representative motion-corrected micrograph out of 3,441 total micrographs, 3D class averages, gold-standard FSC curves of the final 3D reconstruction (resolution cut-off at FSC = 0.143), and the final 3D map (colored according to the local resolution) are shown. 2D class averages were calculated using refined particles that were used for the final reconstruction. 3D class selected for the following analyses are indicated with red dotted boxes.

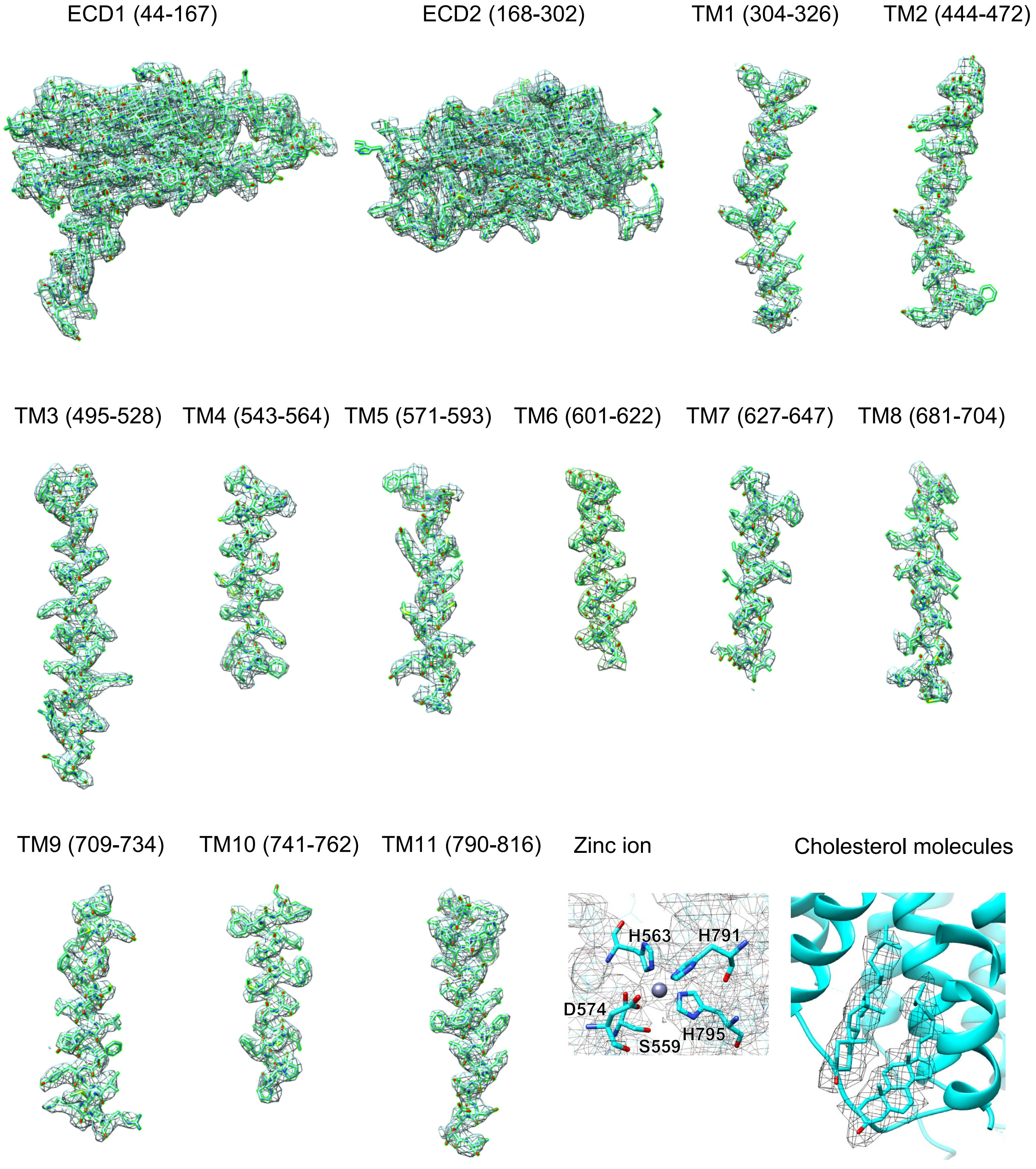

Supplementary Fig. 3 Representative density for the extracellular and transmembrane regions of the cholesterol-bound form of human SIDT1.

Representative cryo-EM density maps of the cholesterol-bound form of human SIDT1 around ECD1, ECD2, each TM helix of human SIDT1, zinc ion and cholesterol molecules are shown.

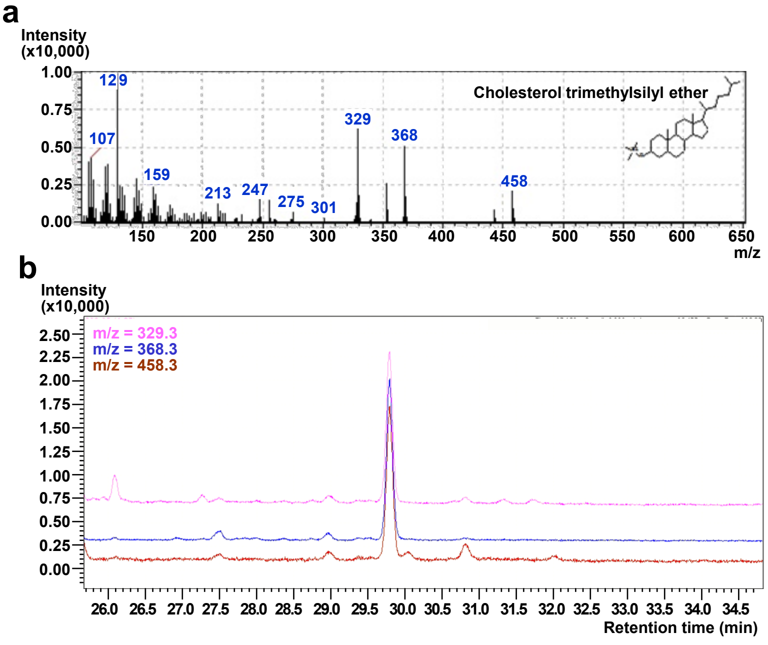

Supplementary Fig. 4 GC-MS identification of cholesterol in purified hSIDT1.

a, The spectrum of cholesterol trimethylsilyl ether.

b, The sample was extracted from purified hSIDT1 and reacted with a trimethylsilylating agent as described in Methods section. GS-MS was conducted by the quantitative analysis of cholesterol using peaks at m/z 329, 368 (fragment ions of cholesterol trimethylsilyl ether) and 458 (precursor ion of cholesterol trimethylsilyl ether). The peak at retention time ~29.8 min was detected as cholesterol.

Human SIDT1 in the presence of 25 bp dsRNA

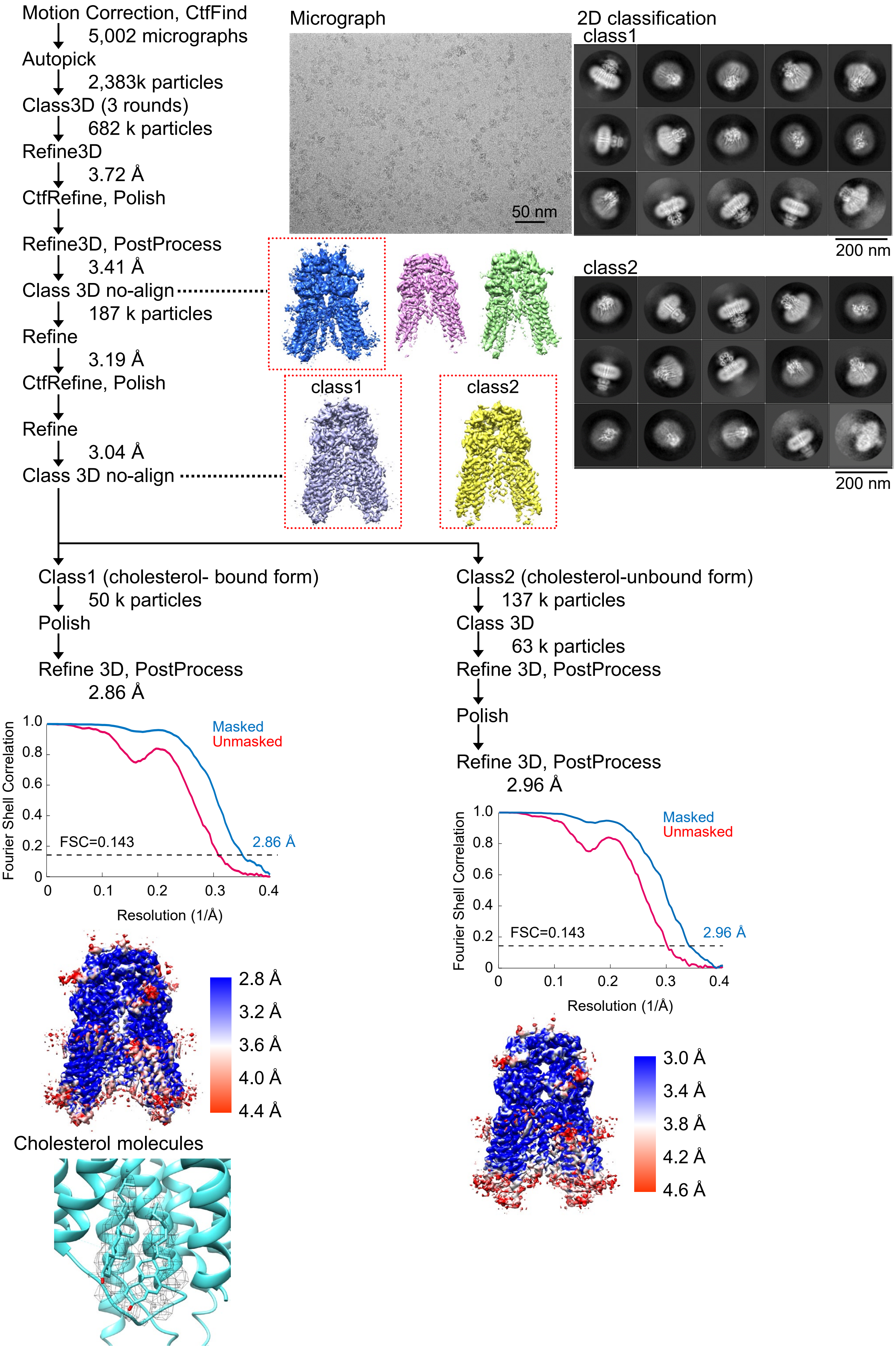

Supplementary Fig. 5 Cryo-EM analysis of human SIDT1 in the presence of 25 bp dsRNA.
Data processing workflow of cryo-EM analysis of human SIDT1 in the presence of 25 bp dsRNA. Representative motion-corrected micrograph out of 5,002 total micrographs, 3D class averages, gold-standard FSC curves of the final 3D reconstruction (resolution cut-off at FSC = 0.143), and the final 3D map (coloured according to the local resolution) are shown. 2D class averages were calculated using refined particles that were used for the final reconstruction. 3D class selected for the following analyses are indicated with red dotted boxes. The density maps of cholesterol bound to hSIDT1 is shown.

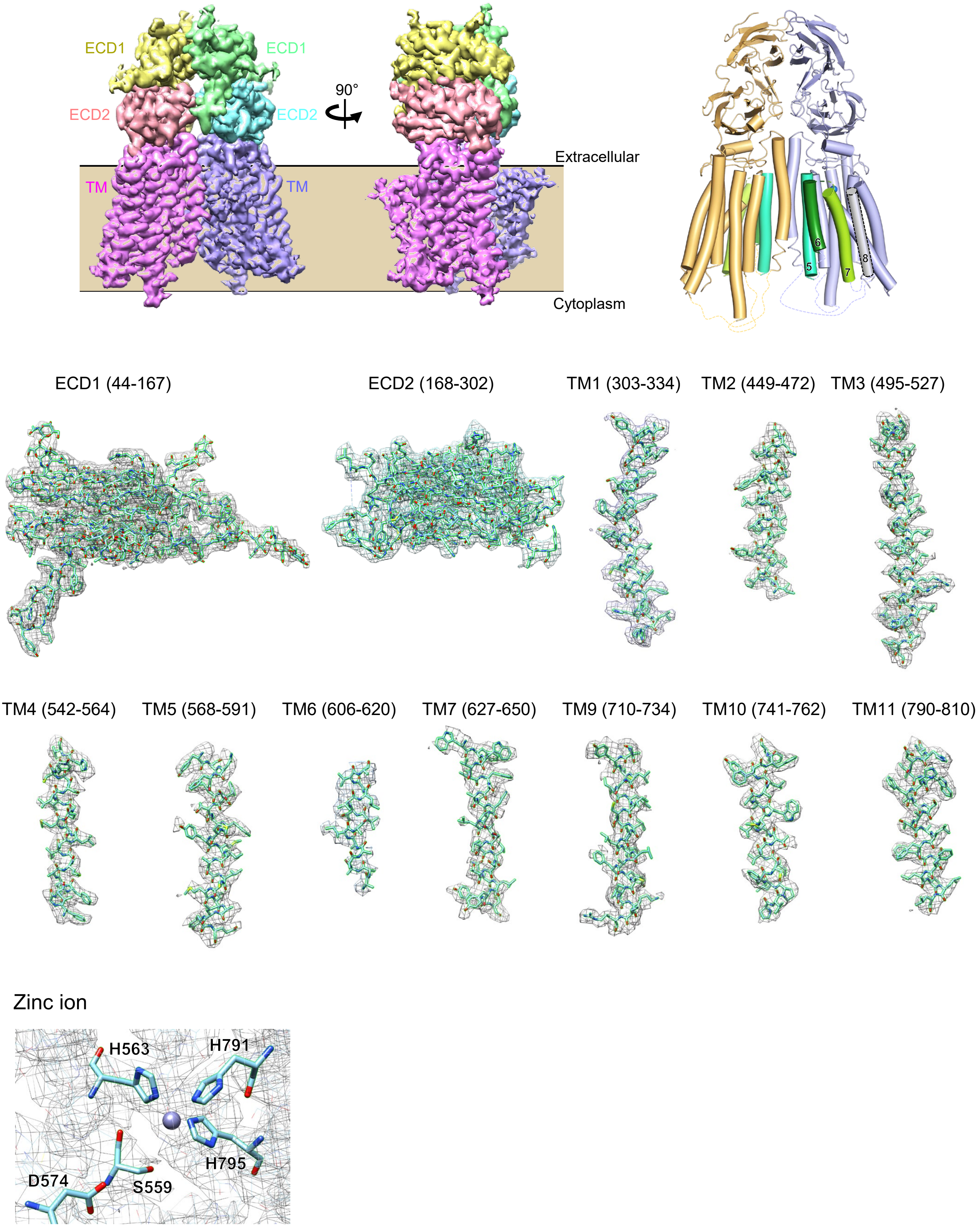

Supplementary Fig. 6 Representative density for the extracellular and transmembrane regions of the cholesterol-unbound form of human SIDT1.

Representative cryo-EM density maps of the cholesterol-unbound form of human SIDT1 around ECD1, ECD2 and each TM helix of human SIDT1 and zinc ion are shown.

Human SIDT1 at pH5.0

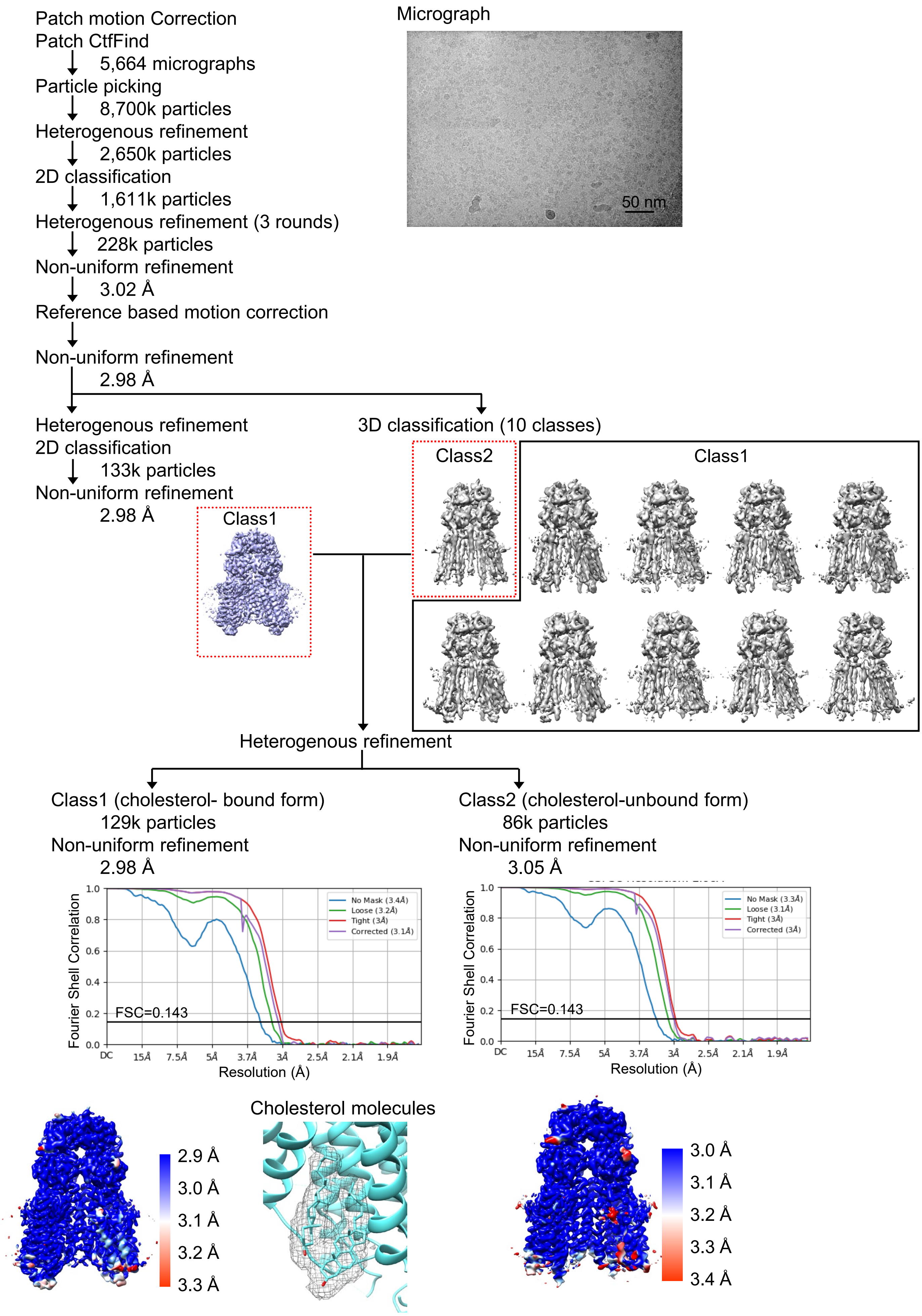

Supplementary Fig. 7 Cryo-EM analysis of human SIDT1 at pH 5.0.

Data processing workflow of cryo-EM analysis of human SIDT1 at pH 5.0. Representative motion-corrected micrograph out of 5,664 total micrographs, 3D class averages, gold-standard FSC curves of the final 3D reconstruction (resolution cut-off at FSC = 0.143), and the final 3D map (coloured according to the local resolution) are shown. The density maps of cholesterol bound to hSIDT1 is shown.

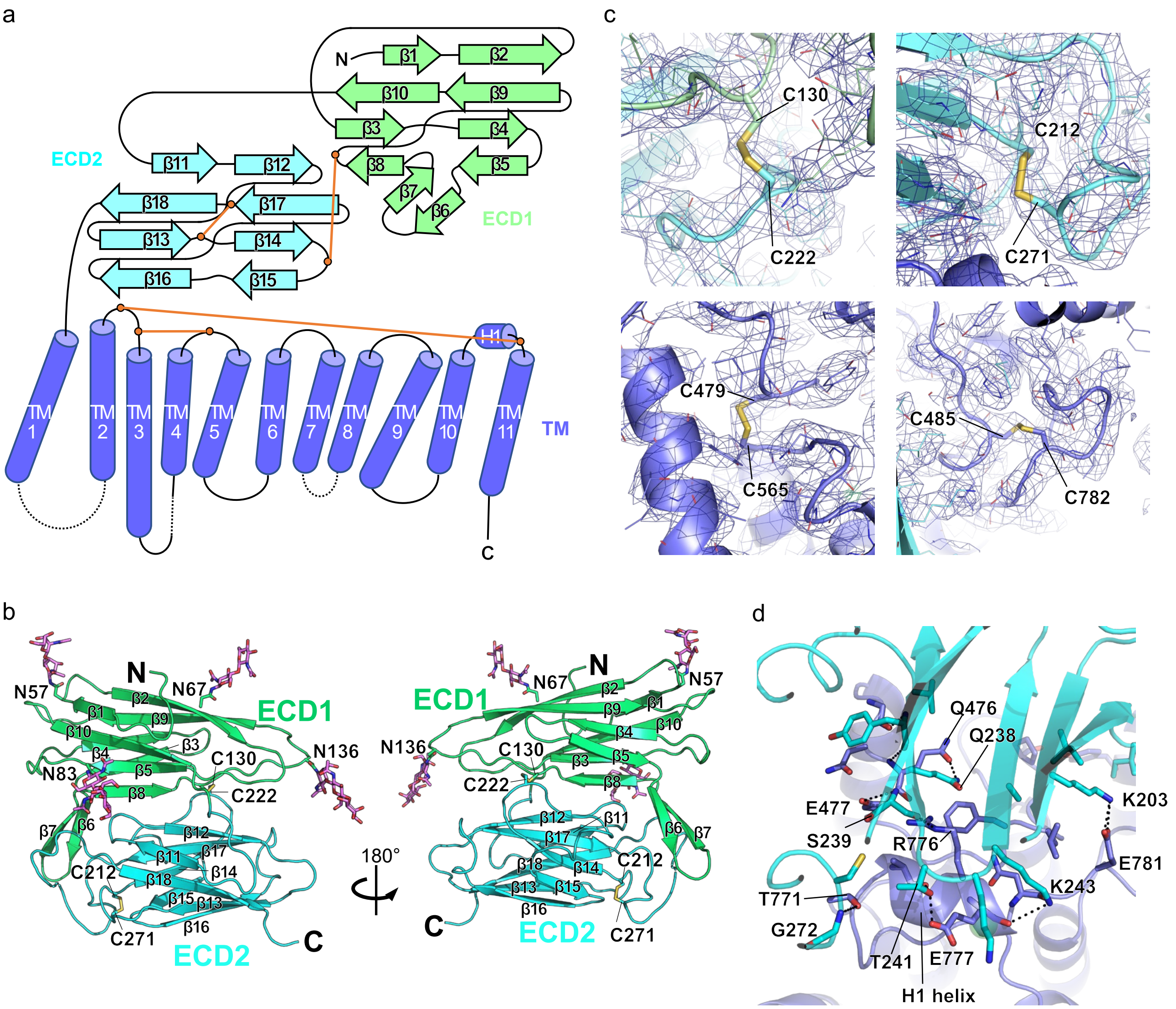

Supplementary Fig. 8 The assembly of extracellular region of SIDT1.

(a) Topology diagram of human SIDT1. Dotted lines indicate disordered regions in the cryo-EM map in the structure of human SIDT1 cholesterol-bound form. The β-strand and α-helix are shown in arrowhead and cylinder, respectively.

(b) Structure of the extracellular region of human SIDT1consists of 2 domains (ECD1 and ECD2) which adopt β-sandwich folds. The extracellular region has four N-glycosylation (Asn57, Asn67, Asn83 and Asn136) and two disulfide bonds (Cys130-Cys222 and Cys212-Cys271). ECD1 and ECD2 are tightly packed with each other.

(c) The cryo-EM densities around disulfide bonds are shown.

(d) The N-terminal extracellular domain is packed with TM segments through the interaction between ECD2 and TM2-TM3/TM10-TM10 loops. Residues that forms hydrogen bond or salt bridge (dotted line) are shown with label. Disulfide bonds are shown as orange lines.

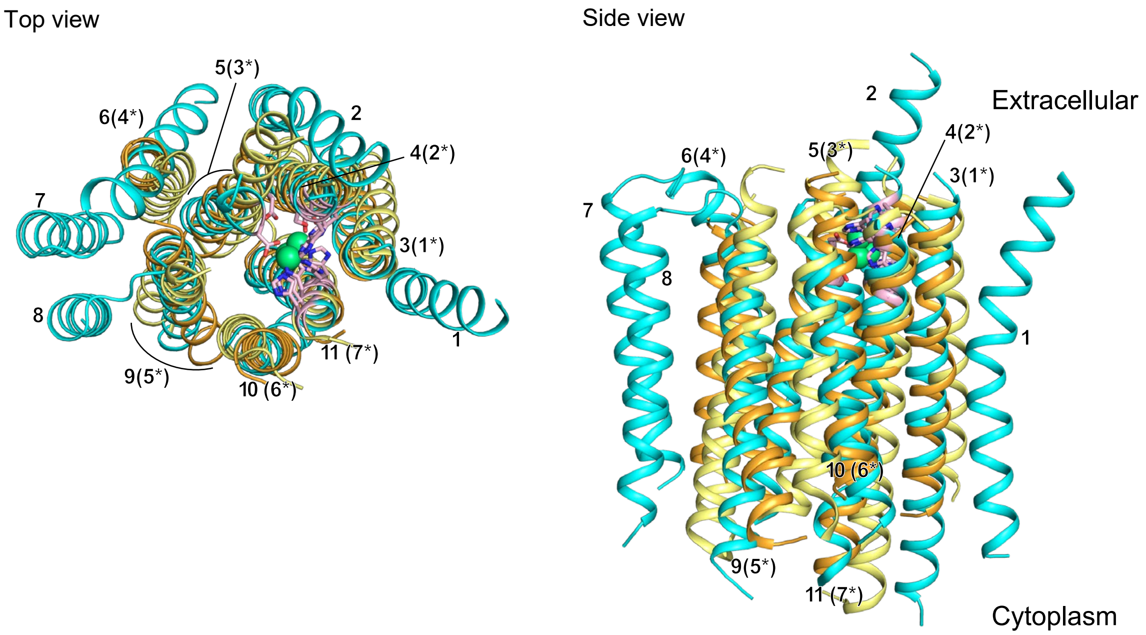

Supplementary Fig. 9 The TM domain of SIDT1 has a structural similarity with ACERs and ADIPORs.

Pairwise structure comparisons between the cholesterol-bound form of SIDT1 and Adiponectin receptor 2 (ADIPOR2, PDB ID: 5LX9) or Alkaline ceramidase 3 (ACER3, PDB: 6G7O) 7 TMs were performed using DALI server[20]. 7 out of 11 TMs in SIDT1 (cyan) were superimposed with all 7 TMs in AIPOR2 (yellow) and ACER3 (orange) with an overall r.m.s. d. of 3.4 Å and 4.5 Å for Cα atoms, respectively. The transmembrane segments with numbering of SIDT1 are indicated, while those of ACER3 and ADIPOR are indicated in parentheses. Extracellular domains and loops are omitted for clarification. Catalytic residues and Zn^{2+} ions are shown in pink stick model and sphere model, respectively.

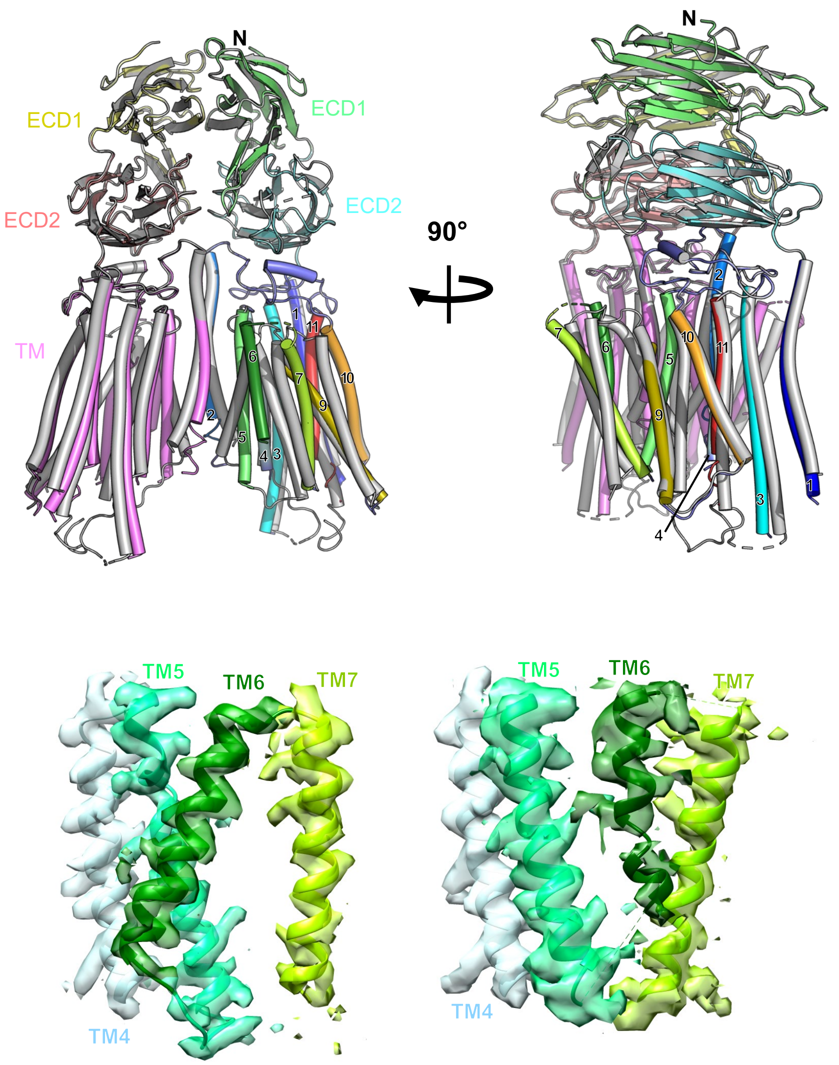

Supplementary Fig. 10 The conformational interconversion of the SIDT1 TM region upon cholesterol binding.

Pairwise structure comparisons between the cholesterol-bound and -unbound form of SIDT1. The cryo-EM density map if TM5, TM6 and TM7 which undergo significant conformation change are shown.

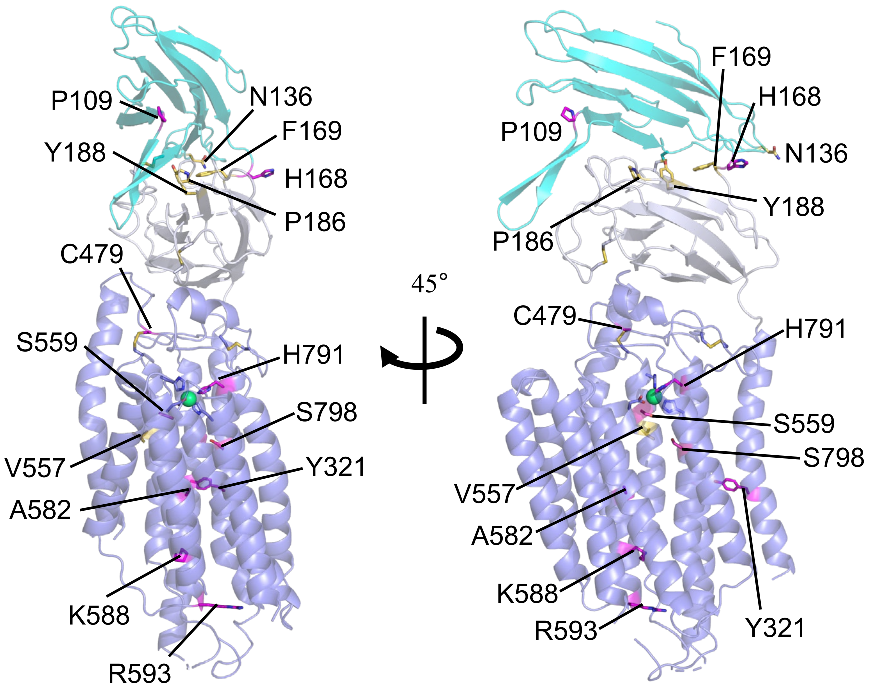

Res.	human	RNAi phenotype	position	properties
M1I	M1	weak	N-terminus	disordered
P108S	P109	strong	ECD1	buried. cis configuration
D130N	N136	weak	ECD1	exposed
R172W	H168	strong	ECD1-ECD2 linker	exposed
A173T	F169	weak	ECD1-ECD2 linker	facing to dimer interface, not involved in dimer interface
P199L	P186	weak	ECD2	facing to dimer interface, not involved in dimer interface
G201E	Y188	weak	ECD2	facing to dimer interface, not involved in dimer interface
G201R	Y188	strong	ECD2	facing to dimer interface, not involved in dimer interface
P328L	Y321	strong	TM1	hydrogen bind with main chain carboxy group of L506 (TM3)
C464Y	C479	strong	TM2-TM3	TM3 loop, disulfide bond with C565
G488E	G503	strong	TM3	buried in the protein. May disturb the protein conformation
G493R	G508	strong	TM3	buried in the protein. May disturb the protein conformation
I534N	V557	weak	TM4	exposed
S536I	S559	strong	TM4	Putative catalytic residue
C559Y	A582	strong	TM5	bulky residue may disturb the TM domain conformation
R565C	K588	strong	TM5	cation-π interaction with Phe545 (TM4)
R570C	R593	strong	TM5	Salt bridge with Glu737
H740Y	H791	strong	TM11	Zn-binding
G747R	S798	strong	TM11	near Zn-binding site

Supplementary Fig. 11 Mapping of mutations that attenuate systemic RNA by SID-1 on the structure of SIDT1.

Mutations that attenuate systemic RNA by SID-1 are mapped on the structure of SIDT1 (upper panel). Mutations of SID-1 and their properties are listed (lower panel).

a

b

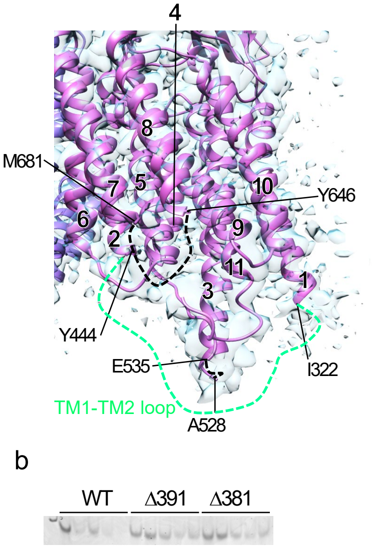

Supplementary Fig. 12 The structure of SIDT1 in the presence of dsRNA.

(a) The cytoplasmic region of hSIDT1 cholesterol-bound form is shown. The TM1-TM2, TM3-TM4 and TM7-8 loops are discontinued, which indicated as dashed lines. The cytoplasmic TM1-TM2 loop is largely disordered in the cryo-EM map (green dashed line).

(b) EMSA assay using full length hSIDT1 or mutant with TM1-2 loop deletion (Δ391: 391-442, Δ381: 381-442). Unshifted band corresponding to unbound dsRNA was monitored to estimate dsRNA binding with SIDT1 since hSIDT1-dsRNA complex less migrated. Increasing concentration of hSIDT1 (1, 2, 3, 4, 5 μM) was incubated with 1 μM of dsRNA and subjected to electrophoresis.

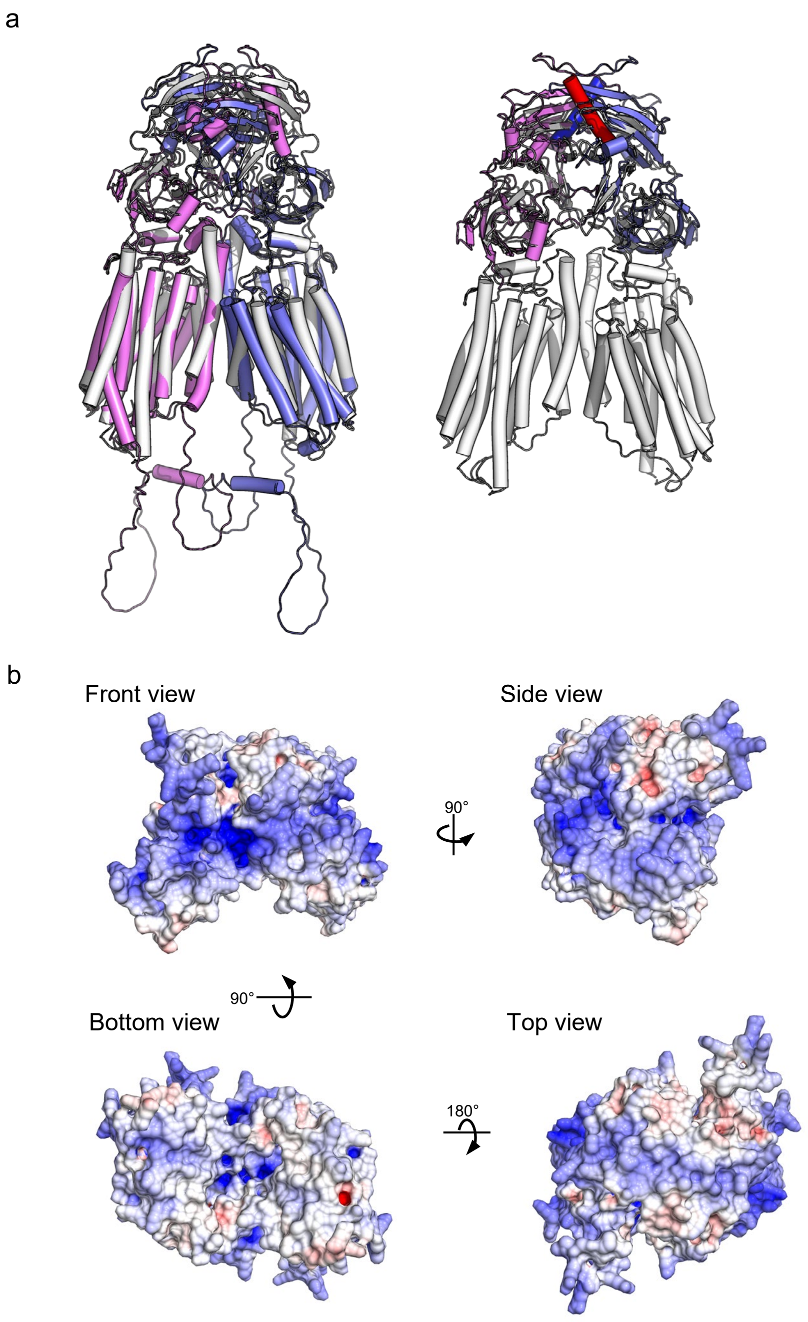

Supplementary Fig. 13 The electrostatic surface potential of predicted structure of C. elegans SID-1.

(a) Two C. elegans SID-1 structures predicted by Alpha-Fold (magenta and purple) were each superimposed on each protomer of hSIDT1 homodimer cholesterol-bound form (white) (left panel). The TM region of SID-1 is relatively fit to that of SIDT1, while the extracellular domain (ECD) is not fitted well and conflicts with the other protomer due to the difference of the orientation between ECD and TM.

On the other hand, when ECDs of two SID-1 structures were each superimposed on each ECD of SIDT1 homodimer (right panel), SID-1 ECDs were well fitted to SIDT1 with less confliction except for the N-terminal helix (red and blue). The N-terminal helix (residue 2-16) is connected to ECD via flexible linker (residues 17-30), which suggests that the orientation of the N-terminal helix is flexible.

(b) The electrostatic surface potential of the ECD dimer model of SID-1 as right panel in (a) is shown in the same orientation as Figure 4a for comparison with SIDT1. The N-terminal 30 residues in SID-1 are omitted in the model for clarity.

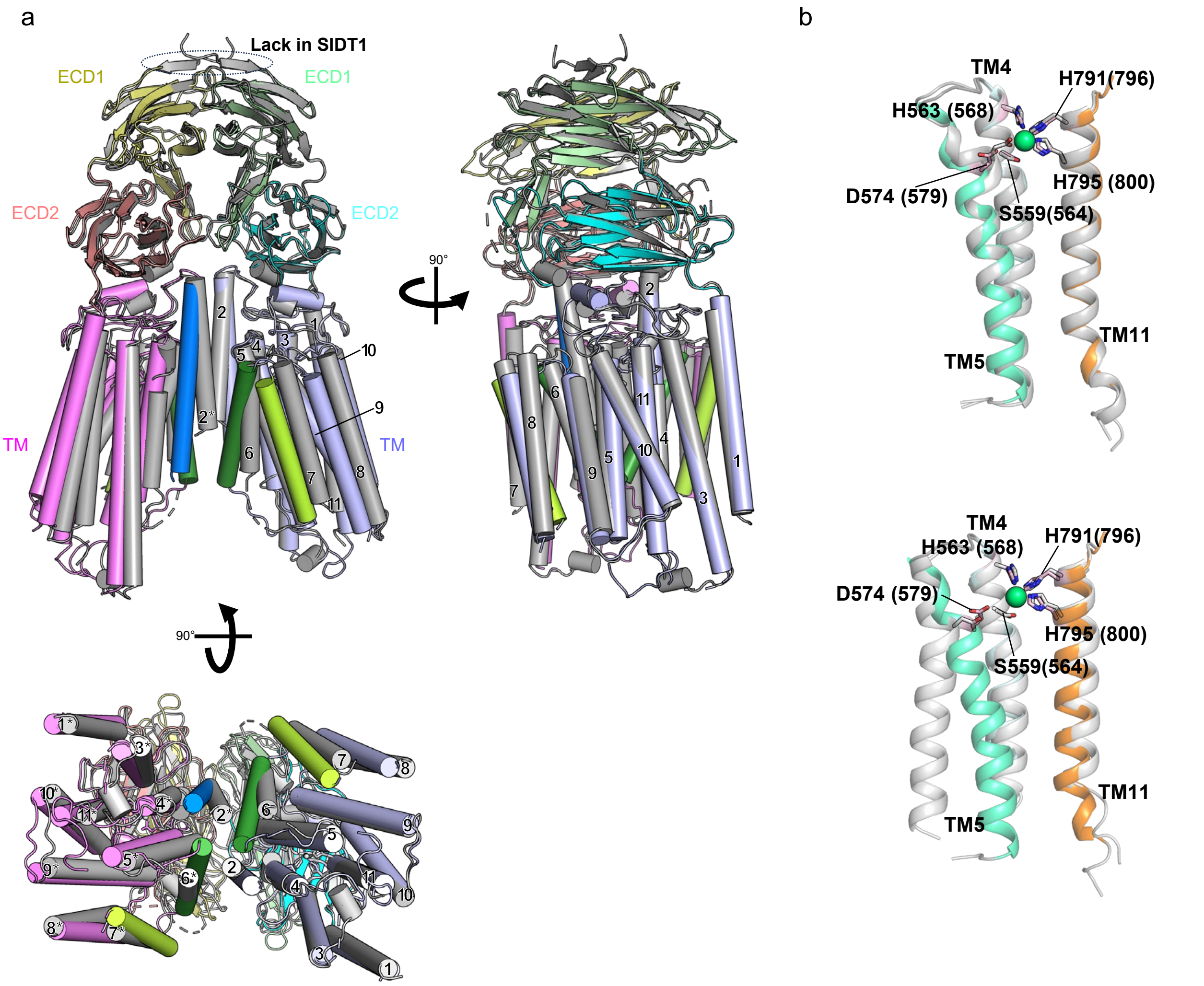

Supplementary Fig. 14 The structural comparison between SIDT1 and SIDT2.

a. Superposition of human SIDT1 (cholesterol-bound form) and human SIDT2 (gray, PDB: 7Y63) are shown. The TM numbers of the superimposed protomer are shown, but * is added to the number of the other protomer for clarity. SIDT1 lacks the first β-strand in ECD1 but has a longer TM2 and TM6 helices. Structural deviation of Cα atoms in TM6, TM7 and TM8 are higher than other regions.

b. Structural comparison focused on the catalytic site. Catalytic residues of human SIDT2 are superimposed on those of human SIDT1 cholesterol-bound form (top panel) or SIDT1 cholesterol-unbound form (bottom panel). Catalytic residues are shown with stick models and the zinc ion is shown as green sphere. Residue numbers of SIDT1 are indicated while those of SIDT2 are indicated in parentheses.

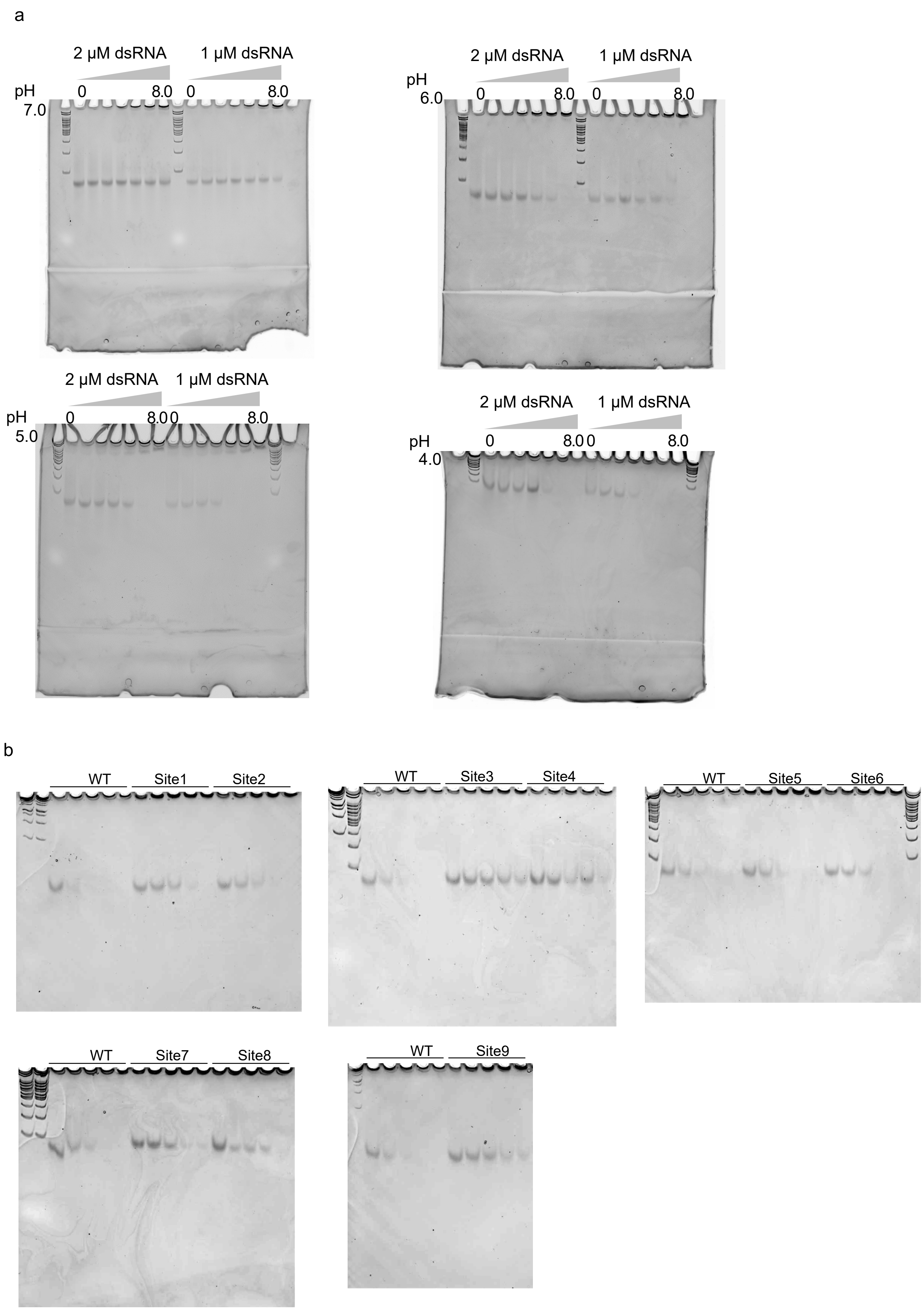
a
2 µM dsRNA
1 µM dsRNA
pH
0
8.0
7.0
6.0
5.0
4.0
b
WT
Site1
Site2
Site3
Site4
Site5
Site6
Site7
Site8
Site9

c

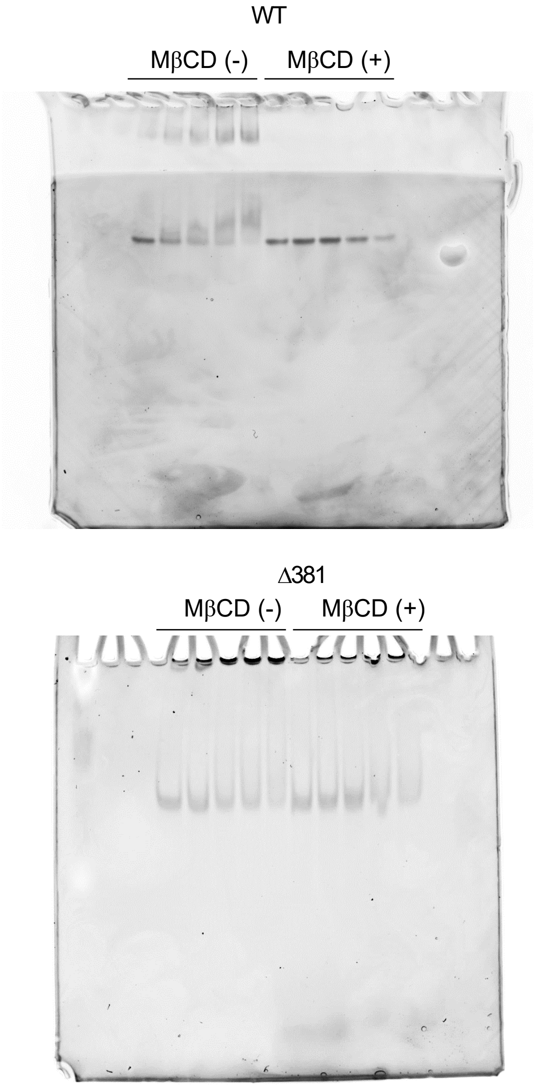

d

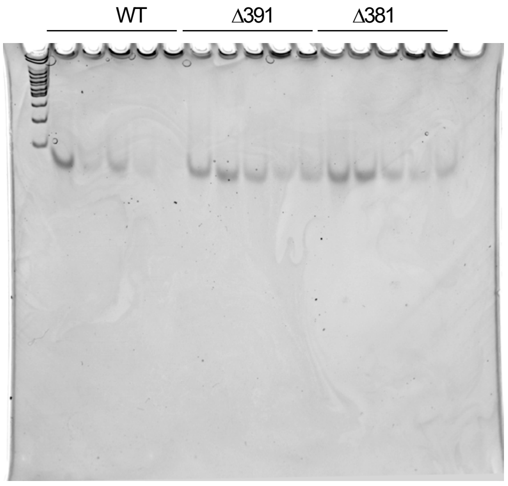

Supplementary Fig. 15 Uncropped gel image of EMSA assay.

Uncropped gel images of Fig. 4b (**a**), Fig. 4c (**b**), Fig. 4f (**c**) and Supplementary Fig. 12b (**d**).

PDB ID	Protein	Expression cell	Detergent for Solubilization	Detergent for Cryo-EM analysis	pH	Conformation of TM region	Binding lipid
8KCW This study	human SIDT1	Expi293F	Digitonin	0.01% GDN	6.0	Closed	Cholesterol
8KCX This study	human SIDT1	Expi293F	Digitonin	0.005% GDN (+dsRNA)	5.0	Open	
This study	human SIDT1	Expi293F	Digitonin	0.005% GDN (+dsRNA)	5.0	Closed	Cholesterol
This study	human SIDT1	Expi293F	Digitonin	0.005% GDN	5.0	Open	
This study	human SIDT1	Expi293F	Digitonin	0.005% GDN	5.0	Closed	Cholesterol
8JUL	human SIDT1	HEK293F	LMNG/CHS	0.02% GDN	7.5	Open	Phosphatidic acid
8JUN	human SIDT1 E555Q	HEK293F	LMNG/CHS	0.02% GDN	7.5	Closed	
8K13	human SIDT1	Sf9	DDM/CHS	0.01% LMNG/ 0.00% CHS	7.5	Open	
8K10	human SIDT2	Sf9	DDM/CHS	0.01% LMNG/ 0.00% CHS	7.5	Closed	
7Y63	human SIDT2	HEK293F	DMNG/CHS	0.006% GDN	7.4	closed	
7Y68	human SIDT2	HEK293F	DMNG/CHS	0.006% GDN (+miRNA)	5.5	Closed	
7Y69	human SIDT2	HEK293F	DMNG/CHS	0.006% GDN	5.5	Closed	

Supplementary Table 1 Structural studies of SIDT1 and SIDT2
Expression cells, detergent used in protein purification procedures and pH for structural studies of SIDT1/2 are summarized. The conformations of TM region in our cholesterol-bound form and cholesterol-unbound form are referred to as Closed and Open, respectively.
