# Supplementary material for: Cryo-EM analysis reveals human SID-1 transmembrane family member 1 dynamics underlying lipid hydrolytic activity
Source: Commun Biol. 2024 May 29;7:664. doi: 10.1038/s42003-024-06346-8 (PMC11137008; doi:10.1038/s42003-024-06346-8)
Supplement: Supplementary file 4 — Reporting Summary [file 42003_2024_6346_MOESM4_ESM.pdf]

nature portfolio

Corresponding author(s): Toshiyuki Shimizu

Last updated by author(s): May 2, 2024

Reporting Summary

Nature Portfolio wishes to improve the reproducibility of the work that we publish. This form provides structure for consistency and transparency in reporting. For further information on Nature Portfolio policies, see our Editorial Policies and the Editorial Policy Checklist.

Statistics

For all statistical analyses, confirm that the following items are present in the figure legend, table legend, main text, or Methods section.

n/a	Confirmed	
☐	☒	The exact sample size (*n*) for each experimental group/condition, given as a discrete number and unit of measurement
☐	☒	A statement on whether measurements were taken from distinct samples or whether the same sample was measured repeatedly
☐	☒	The statistical test(s) used AND whether they are one- or two-sided<br>*Only common tests should be described solely by name; describe more complex techniques in the Methods section.*
☒	☐	A description of all covariates tested
☐	☒	A description of any assumptions or corrections, such as tests of normality and adjustment for multiple comparisons
☐	☒	A full description of the statistical parameters including central tendency (e.g. means) or other basic estimates (e.g. regression coefficient) AND variation (e.g. standard deviation) or associated estimates of uncertainty (e.g. confidence intervals)
☒	☐	For null hypothesis testing, the test statistic (e.g. *F*, *t*, *r*) with confidence intervals, effect sizes, degrees of freedom and *P* value noted<br>*Give P values as exact values whenever suitable.*
☒	☐	For Bayesian analysis, information on the choice of priors and Markov chain Monte Carlo settings
☒	☐	For hierarchical and complex designs, identification of the appropriate level for tests and full reporting of outcomes
☒	☐	Estimates of effect sizes (e.g. Cohen's *d*, Pearson's *r*), indicating how they were calculated

Our web collection on statistics for biologists contains articles on many of the points above.

Software and code

Policy information about availability of computer code

Data collection	Cryo-EM data were collected with Serial EM 3.8.
Data analysis	MotionCor2 (implemented in RELION), CTFFIND4.1 (implemented in RELION), RELION (3.1), Chimera (1.16), COOT (0.9.8), PHENIX (1.20), cryoSPARC (v4.4)

For manuscripts utilizing custom algorithms or software that are central to the research but not yet described in published literature, software must be made available to editors and reviewers. We strongly encourage code deposition in a community repository (e.g. GitHub). See the Nature Portfolio guidelines for submitting code & software for further information.

Data

Policy information about availability of data

All manuscripts must include a data availability statement. This statement should provide the following information, where applicable:

- Accession codes, unique identifiers, or web links for publicly available datasets
- A description of any restrictions on data availability
- For clinical datasets or third party data, please ensure that the statement adheres to our policy

The cryo-EM maps and related structure coordinate of hSIDT1 cholesterol-unbound form and cholesterol-bound form have been deposited in the EMDB and PDB under accession codes EMD-37113, EMD-37112 and PDB: 8KCX and 8KCW, respectively.

Field-specific reporting

Please select the one below that is the best fit for your research. If you are not sure, read the appropriate sections before making your selection.

☒ Life sciences ☐ Behavioural & social sciences ☐ Ecological, evolutionary & environmental sciences

For a reference copy of the document with all sections, see nature.com/documents/nr-reporting-summary-flat.pdf

Life sciences study design

All studies must disclose on these points even when the disclosure is negative.

Sample size	Cryo-EM sample size are indicated in Supplementary Fig. 2, 5. 7
Data exclusions	Cryo-EM images were excluded based on CTF max resolution parameters. Picked particles were excluded after 2D and 3D classification procedures using cryoSPARC and RELION 3.1 software. Bad class averages were excluded manually based on general appearance, estimated resolutions and map quality. These procedures are general practice in the field of cryo-EM single particle analysis.
Replication	All attempts at replication were successful.
Randomization	Randomization was performed when calculating Fourier-shell correlation of half maps and R-free values.
Blinding	n/a

Reporting for specific materials, systems and methods

We require information from authors about some types of materials, experimental systems and methods used in many studies. Here, indicate whether each material, system or method listed is relevant to your study. If you are not sure if a list item applies to your research, read the appropriate section before selecting a response.

Materials & experimental systems

n/a	Involved in the study	
☒	☐	Antibodies
☐	☒	Eukaryotic cell lines
☒	☐	Palaeontology and archaeology
☒	☐	Animals and other organisms
☒	☐	Human research participants
☒	☐	Clinical data
☒	☐	Dual use research of concern

Methods

n/a	Involved in the study	
☒	☐	ChIP-seq
☒	☐	Flow cytometry
☒	☐	MRI-based neuroimaging

Eukaryotic cell lines

Policy information about cell lines

Cell line source(s)	Expi293F and Sf9 cells were purchased from ThermoFisher Scientific (cat# A14635 and 11496015).
Authentication	Expi293F and Sf9 cells were validated by the manufacturer.
Mycoplasma contamination	All cell lines tested negative for mycoplasma contamination.
Commonly misidentified lines (See ICLAC register)	n/a
